# Constitutive *AtRD29A* expression reveals divergent *BpCBF3* regulatory mechanisms in birch

**DOI:** 10.1111/tpj.71121

**Published:** 2026-09-25

**Authors:** Mengyan Ge, Xinyu Wang, Lili Hou, Guifeng Liu, Zhimin Zheng

**Affiliations:** ^1^ State Key Laboratory of Tree Genetics and Breeding College of Forestry, Northeast Forestry University Harbin 150040 China; ^2^ The Center for Basic Forestry Research College of Forestry, Northeast Forestry University Harbin 150040 China

**Keywords:** *Betula platyphylla*, *CBF/DREB1*, cold stress, *AtRD29A promoter*, constitutive expression, transcriptional regulation

## Abstract

Abiotic stress resistance in plants is often associated with growth retardation. To address this limitation, stress‐inducible promoters can be used to generate plants that exhibit both rapid growth and high stress resistance. *RD29A* (Response to Desiccation 29A) is a widely used stress‐inducible promoter in *Arabidopsis thaliana* (*AtRD29A*) that can be activated by multiple factors including NaCl, ABA, and low temperature. Specifically, under cold stress, it is induced through the response to C‐repeat/DREB binding factors (*CBFs*). In this study, we identified five *CBF3* homologous genes—*BpCBF3e*, *BpCBF3b*, *BpCBF3d*, *BpCBF3c*, and *BpCBF3a—*in birch (*Betula platyphylla*), but did not detect an *RD29A* homolog. We proposed *BpCBF3d* to be the functional *CBF* because its expression is strongly regulated by circadian rhythm and cold stress, and it activates downstream cold response marker genes. To confirm this hypothesis, we performed Y1H assays and generated *BpCBF3d* overexpression lines driven by two different promoters—*p35S* and *pRD29A*. Notably, we found that *BpCBF3d* did not bind to the *AtRD29A* promoter, whereas *BpCBF3e*, *BpCBF3b*, *BpCBF3c*, and *BpCBF3a* bound strongly, resulting in constitutive expression of heterologous *AtRD29A* in birch. This study provides new molecular tools for breeding forest trees with enhanced resilience to environmental change.

## Significance Statement

Five *AtCBF3*‐homologous genes are tandemly arranged on chromosome 11 in birch (*Betula platyphylla*), among which *BpCBF3d* is the key C‐repeat/DREB binding factor (CBF) primarily responsible for cold‐responsive function. The gene directly downstream of CBF, *AtRD29A—*which is found exclusively in Brassicaceae*—*exhibits a shift from inducible to constitutive expression when heterologously expressed in birch.

## INTRODUCTION

Forests constitute a vital component of terrestrial ecosystems and serve as key regulators of material cycling and energy flow within natural ecosystems. However, in recent years, severe climate change has significantly impacted the health and stability of forests worldwide (Bennett et al., 2015; Li, Piao, et al., 2020). Previous research on the holistic responses of forest ecosystems to environmental stressors has predominantly focused on high temperature and drought conditions, while limited attention has been given to the mechanisms that enable trees to survive prolonged, harsh winters in high‐latitude regions. Consequently, our understanding of the molecular mechanisms through which forest trees respond to environmental stressors remains limited.

Plant growth and development are often affected by a wide range of stress factors, including both biotic and abiotic stressors (Zhu, 2016). With the accelerating pace of global climate change, abiotic stresses such as osmotic stress, salinity, abnormal temperature, and drought are exerting increasingly severe impacts on forest trees (Agarwal et al., 2006). Among these stresses, low‐temperature stress represents one of the most significant climate challenges for trees in high‐latitude forest ecosystems. During the long‐term evolutionary process, plants have gradually formed a response mechanism to a variety of stresses. The C‐repeat/DREB binding factor (CBF)‐cold‐regulated (COR) signal transduction pathway is the most widely studied for low‐temperature stress, and the *CBF* gene family plays a crucial role in plant resistance to low‐temperature stress (Ito et al., 2006; Lata & Prasad, 2011; Mizoi et al., 2012; Warren, 1998).

The *CBF* gene family belongs to the AP2/ERF superfamily, which is one of the largest TF families involved in the regulation of growth, development, and biotic and abiotic stress responses in plants (Mizoi et al., 2012; Wessler, 2005). In Arabidopsis, three members of the *CBF* family are involved in plant responses to low‐temperature stress, namely *CBF1/DREB1B*, *CBF2/DREB1C*, and *CBF3/DREB1A*. These transcription factors can bind to cis‐acting elements of the CRT/dehydration‐responsive element (DRE) in the downstream gene promoter (for example, *AtRD29A*), thereby regulating the expression of downstream genes related to the plant hypothermic stress response (Sakuma et al., 2002). Notably, no CBF/DREB1 protein has been found in algae, mosses, ferns, or gymnosperms, with members of this protein family existing only in angiosperms (Li, Chen, et al., 2020). Several studies have demonstrated increased accumulation of dehydrin transcripts or proteins in woody plants during the coldest months. Similar to Arabidopsis, the cold‐inducible dehydrin genes in woody plants contain CRT elements in their promoters (Artlip et al., 1997; Rinne et al., 1998; Welling et al., 2004; Wisniewski et al., 2006). The birch dehydrin gene *BpLTI36* is cold‐inducible and contains several CRT elements within its promoter. Reporter gene‐promoter fusion analyses have shown that Arabidopsis CBFs can recognize birch promoter elements and activate reporter genes, suggesting that birch also possesses a functional CBF regulon (Puhakainen et al., 2004).

Although CBFs target multiple downstream genes, *AtRD29A* in Arabidopsis is one of the most extensively studied stress‐inducible promoters, which can be activated by diverse environmental stress factors, including low temperature, salinity, drought, and abscisic acid (ABA) (Bihmidine et al., 2013; Maruyama et al., 2012). *AtRD29A* promoter has been widely used to mitigate the negative effects of genes whose overexpression causes growth retardation or reduces yield and has been identified as the most effective stress‐inducible promoter among various promoters (Feng et al., 2011; Kasuga et al., 1999; Zhao, Chan, et al., 2016). In potato, heterologous overexpression of Arabidopsis *RD29A::DREB1A* significantly increased the cold tolerance of transgenic potato (Behnam et al., 2007). Similarly, overexpression of Arabidopsis *RD29A::DREB1A* in tomato significantly improved drought tolerance (Rai et al., 2013); similar results were observed in soybean (Bihmidine et al., 2013; de Paiva Rolla et al., 2014). However, the use of inducible promoters to drive resistance genes has not yet been reported in woody plants.

In this study, we identified five *CBF*s (*CBF3a–e*) and generated transgenic birch lines overexpressing these genes under the control of the *35s* and *AtRD29A* promoters. Notably, heterologous expression of the Arabidopsis *RD29A* promoter in birch resulted in constitutive activity rather than stress‐induced expression. Yeast one‐hybrid (Y1H) analysis revealed divergent regulatory mechanisms: *BpCBF3e*, *BpCBF3b*, *BpCBF3c*, and *BpCBF3a* exhibited high affinity binding to the *AtRD29A* promoter, whereas *BpCBF3d*, identified as a functional *CBF* ortholog, failed to bind. This differential binding explains the constitutive activation of *AtRD29A* in birch, in contrast to its stress‐responsive behavior in Arabidopsis.

## RESULTS

### Identification of *CBF/DREB1* homologs in birch and other angiosperms

An HMMER model was constructed using protein sequences of the six *CBF* genes from *Arabidopsis thaliana* as templates (AT4G25490, AT4G25470, AT4G25480, AT5G51990, AT1G12610, AT1G63030). The results indicated that all AtCBF proteins contained a conserved AP2 domain. This model was used to perform homology searches of the birch genome, leading to the identification of 145 *AP2* family members. A phylogenetic tree constructed using these 145 birch *AP2* members together with the Arabidopsis *CBF*s revealed 12 birch genes closely related to Arabidopsis *CBF*s. Subsequent motif analysis and amino acid sequence similarity assessment of these 18 candidate genes resulted in the identification of five birch *CBF3* homolog genes (Figure S1). Based on amino acid similarity to Arabidopsis *CBF*s and their chromosomal locations, these genes were designated *BpCBF3e*, *BpCBF3b*, *BpCBF3d*, *BpCBF3c*, and *BpCBF3a*.

**Figure 1:**
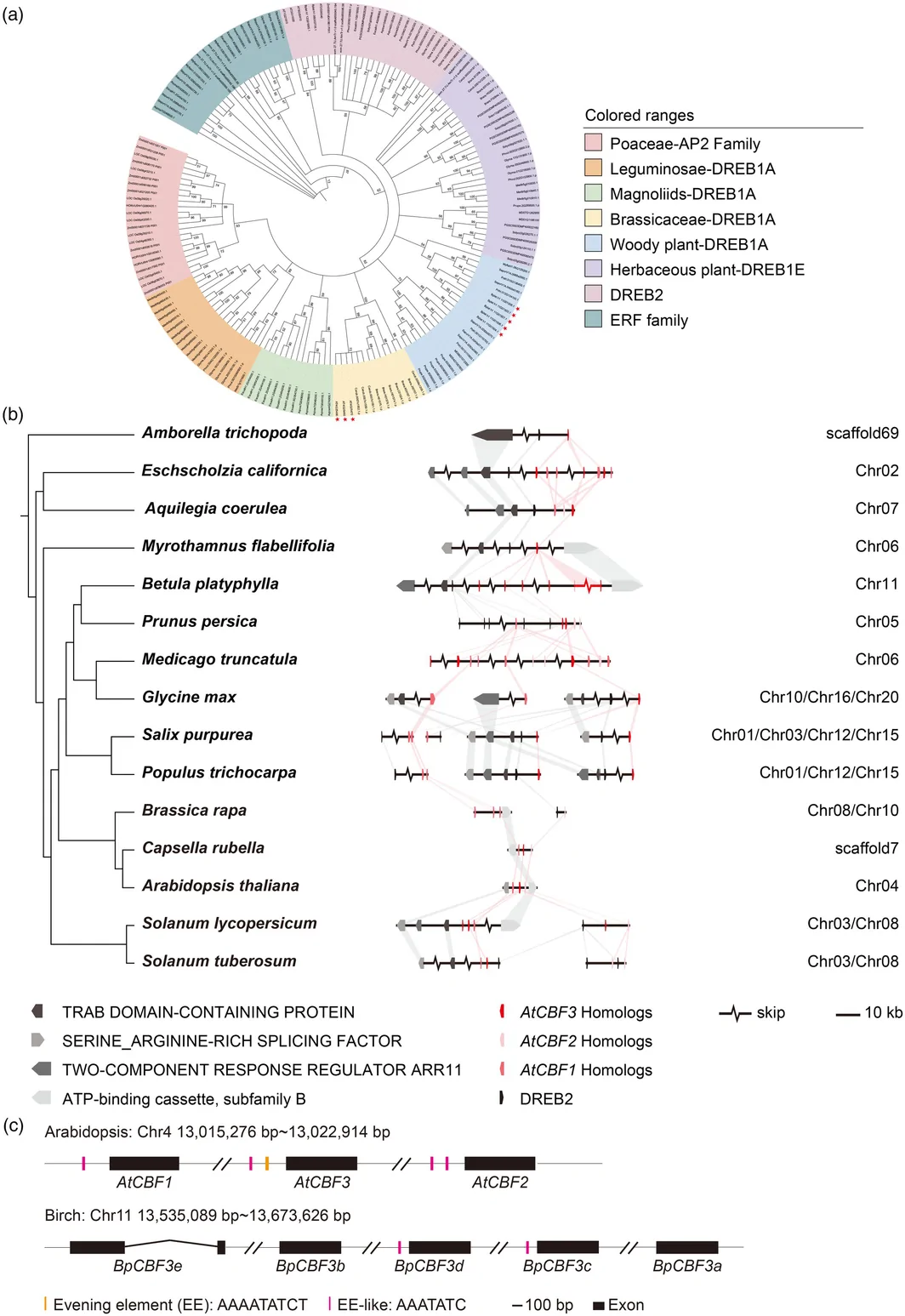
*BpCBF3d* and *BpCBF3c* are potential functional C‐repeat/DREB binding factor (*CBF*) homologs in birch. (a) Phylogenetic tree of *CBF/DREB1* genes from 20 plant species. Bootstrap values were calculated with 1000 replicates using MEGA X. The genes fell into eight major clades, each highlighted in a distinct color. *CBF*s from Arabidopsis and birch are prominently indicated by red star symbols. (b) Evolutionary analysis of *CBF/DREB1* homologs. Selected species containing *DREB1A* from (a) were used to construct an evolutionary timeline (left). The genomic locations of CBF homologs and their neighboring genes are shown on chromosomes. The three *CBF* homologs are depicted in different shades of red, while adjacent genes are shown in varying shades of dark or gray. Chromosome numbers are indicated on the right. Arrow directions indicate transcriptional orientation. Genes sharing the same color are annotated as identical homologs. (c) Genetic structure of *CBF*s in Arabidopsis and birch. The three Arabidopsis *CBF*s and the five birch *CBF3* genes are arranged in tandem repeats on chromosomes, all with the same transcriptional orientation. Each of the three Arabidopsis *CBF*s contains one or two evening element (EE) or EE‐like motifs in its promoter region. In contrast, only *BpCBF3d* and *BpCBF3c* from birch each possess one EE‐like motif in their promoter region.

We employed the same approach to identify 163 *CBF/DREB1* genes from 19 additional species. Gene annotation revealed that these genes could be classified into eight clades, five of which were annotated as *DREB1*, the clade containing functionally active *CBF* genes in plants. Within these five *DREB1* clades, magnoliids, Leguminosae, Brassicaceae, woody plants, and Poaceae species clustered separately, indicating conservation of *CBF* genes at the family level. The remaining three clades were annotated as members of the *AP2* superfamily but lacked conservation across species (Figure 1a; Table S1).

### Evolutionary dynamics of *CBF* in angiosperms

To elucidate the evolutionary dynamics of *CBF* genes, we performed divergence time analysis on the 20 selected species and systematically renamed the *DREB1*‐annotated genes clustered in the phylogenetic tree according to their sequence similarity to *AtCBF1*, *AtCBF2*, and *AtCBF3* (Table S2). A comprehensive analysis of chromosomal gene neighborhoods revealed that most species maintained conserved synteny around *CBF* homologs, which were consistently flanked by functionally related genes, including *AP2/ERF* superfamily members (particularly *ERF* homologs), TRAB domain‐containing proteins, serine/arginine‐rich splicing factors, the two‐component response regulator ARR11, and ATP‐binding cassette subfamily B transporters. This remarkable conservation of genomic architecture surrounding *CBF/DREB1* loci across diverse angiosperms suggests strong evolutionary constraints and functional coordination of this important gene family throughout plant evolution (Figure 1b).

These findings suggest that during the earliest stages of angiosperm evolution, the most ancient flowering plant *Amborella trichopoda* contained only a single *CBF3* homolog located adjacent to an *ERF* gene on the same chromosome. In contrast, the herbaceous species *Aquilegia coerulea* and *Eschscholzia californica* have evolved both *CBF1* and *CBF2* homologs, establishing an initial pattern of tandemly arranged *CBF* gene clusters on the chromosomes. Notably, *Myrothamnus flabellifolia*, another primitive magnoliid similar to *A. trichopoda*, also retains only a single *CBF3* homolog. Furthermore, *M. flabellifolia* is the only known woody resurrection angiosperm with remarkable stress tolerance, further supporting the dominant functional role of *CBF3* within the *CBF/DREB1* gene family. During later evolutionary diversification, some species developed distinct *CBF* homologs distributed across multiple chromosomes (for example, *Malus domestica*, *Glycine max*, *Phaseolus vulgaris*, *Populus trichocarpa*, *Salix purpurea*, *Solanum lycopersicum*, and *Solanum tuberosum*), whereas others formed chromosomal clusters of tandemly duplicated *CBF* genes (for example, *Prunus persica* and *Medicago truncatula*). A unique genomic pattern was observed in birch, where five tandemly arranged *CBF3* genes were identified on chromosome 11. This genomic configuration possibly contributes to the exceptional stress tolerance observed in birch and to its widespread distribution as a pioneer species in high‐latitude cold environments. In contrast, the more recently evolved *Brassicaceae* species exhibited a simplified and conserved gene organization, with only three genes (*CBF1*, *CBF2*, and *CBF3*) forming tandem clusters.

Notably, our parallel evolutionary analysis of *DREB2* homologs (using *AtDREB2A* as a reference) revealed stronger evolutionary conservation than that observed for *CBF* genes. From *A. trichopoda* to Arabidopsis. *DREB2A* homologs consistently maintained co‐linear genetic linkage with ABSCISIC ACID RECEPTOR (PYL) and LACCASE‐12 genes in the same transcriptional orientation, suggesting strong functional constraints governing this genomic neighborhood (Figure S3).

### Characterization of the five *CBF3*s in birch

In summary, functional *CBF* genes (*CBF1*, *CBF2*, and *CBF3*) originating from the *AP2* gene family were duplicated in tandem to form gene clusters on chromosomes during angiosperm evolution. Among all species examined, birch uniquely possesses five cold‐responsive *CBF* genes, all identified as *AtCBF3* homologs. These genes were systematically named *BpCBF3e*, *BpCBF3b*, *BpCBF3d*, *BpCBF3c*, and *BpCBF3a*, based on their chromosomal arrangement. Similar to Arabidopsis, all five *CBF3* genes in birch are tandemly arrayed on the same chromosome. Subcellular localization analysis revealed that, consistent with AtCBF3, all five BpCBF3 proteins are localized in the nucleus (Figure S4). However, only *BpCBF3d* and *BpCBF3c* contain the conserved evening element‐like (EE‐Like) motif in their promoter regions, identical to the regulatory motif found in *AtCBF3* (Dong et al., 2011; Kidokoro et al., 2022) (Figure 1c). Therefore, we propose that *BpCBF3d* and *BpCBF3c* represent the primary functional *CBF* genes in birch.

**Figure 2:**
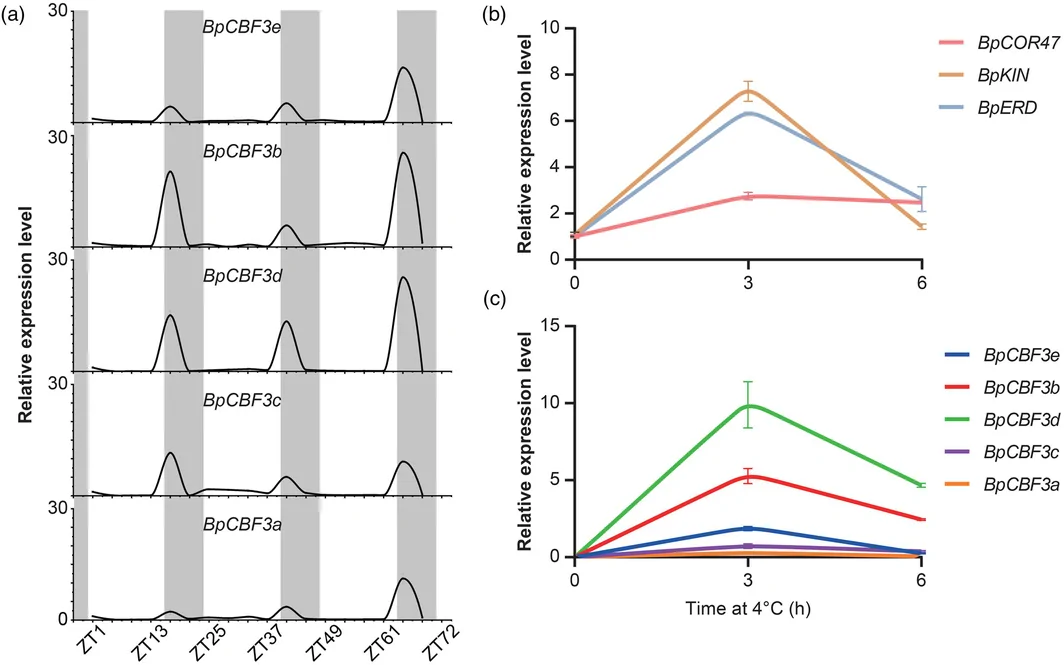
Expression of *BpCBF3*s in response to circadian rhythm and cold stress. (a) Circadian rhythm expression analysis of five *BpCBF3*s in wild‐type (WT) leaves under 16 h light:8 h dark cycles. ZT, Zeitgeber time. White background, light; gray background, dark. (b, c) Cold‐induced expression of COR marker genes (b) and *BpCBF3*s (c). WT plants grown at 25°C for 1 month were transferred to 4°C and sampled at 0, 3, and 6 h. Transcript levels were measured by quantitative reverse transcriptase‐polymerase chain reaction (qRT‐PCR). Values are means ± SD from three independent biological replicates.

To validate the functions of *BpCBF3d* and *BpCBF3c* in birch, we analyzed the expression patterns of all five *CBF3* genes. Circadian rhythm analysis revealed that although the EE‐Like motif was identified only in the promoter regions of *BpCBF3d* and *BpCBF3c*, all five *BpCBF3* genes exhibited significant circadian oscillations (Figure 2a). Among these genes, *BpCBF3d* displayed relatively the greatest oscillation amplitude and the most stable period, which may suggest that it serves as a relatively core functional gene within this family. All *BpCBF3* genes maintained low expression during the daytime, with transcript levels beginning to significantly increase around 20:00, peaking at approximately 24:00, and then gradually declining thereafter. This expression pattern is consistent with typical circadian‐regulated genes, indicating that the expression of the *BpCBF3* gene family is possibly regulated by the endogenous circadian clock.

To further assess their cold responsiveness, wild‐type (WT) birch seedlings were subjected to 4°C treatment. The effectiveness of cold exposure was verified by monitoring the responses of three classic *COR* genes in birch (Figure 2b). All *BpCBF3* genes exhibited rapid upregulation within 3 h of cold treatment, confirming their capacity for rapid response to low‐temperature stress. However, significant differences in response intensity were observed among family members: *BpCBF3d* exhibited the most pronounced induction pattern, with the magnitude of expression change far exceeding that of other homologous genes. Although the remaining four *BpCBF3* genes also responded to low temperatures, the increases in their expression were relatively weaker (Figure 2c). Therefore, it is possible that *BpCBF3d* serves as the most prominent functional *CBF* gene among its homologs in birch.

### Complementation analysis of *BpCBF3*s in Arabidopsis *cbf3* mutant

**Figure 3:**
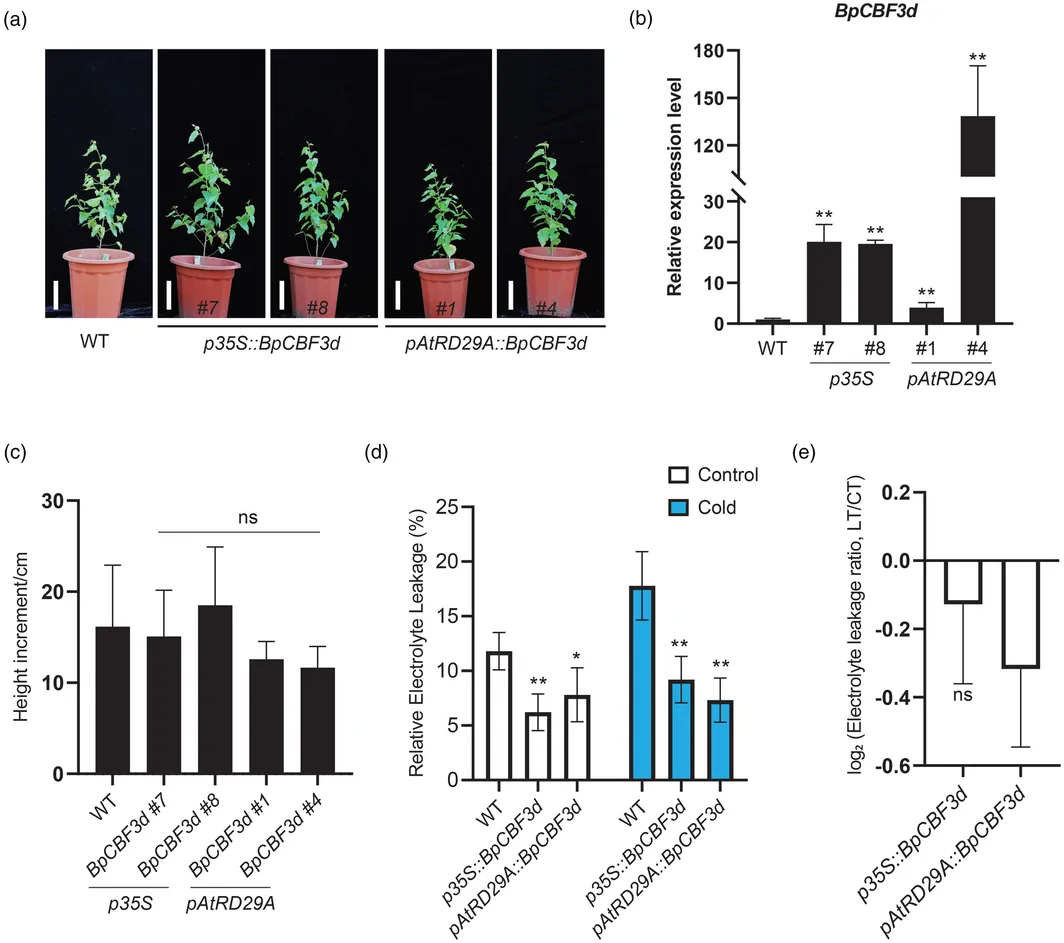
The overexpression of *BpCBF3d* does not affect the growth in birch. (a) Phenotypes of *p35S::BpCBF3d* and *pAtRD29A::BpCBF3d* plants compared with wild type (WT). Plants were grown in soil for 1 year. Bar = 20 cm. (b) Relative gene expression levels of *BpCBF3d* driven by *p35S* and *pAtRD29A* in the field. Transcript levels were determined by qRT‐PCR. Error bars represent mean ± SD from three biological replicates. Asterisks indicate significant differences (*P < 0.05, **P < 0.01; Student's *t*‐test) between transgenic lines and WT. (c) Height increment of *BpCBF3d* driven by *p35S* and *pAtRD29A* in the field. Plants were transferred to soil in September of the first year. Tree height was measured in August and September of the second year, and the increment was calculated as the difference between the two measurements. Error bars represent mean ± SD from three biological replicates. ‘ns’ indicates no significant difference (*P* ≥ 0.05; Student's *t*‐test) between transgenic lines and WT. (d) Electrolyte leakage of WT and *BpCBF3d* overexpression lines under control and cold treatment conditions. Electrolyte leakage was calculated as (S1/S2) × 100, where S1 and S2 represent the conductivity before and after autoclaving, respectively. Data are shown as mean ± SD (*n* = 3 biological replicates). Significant differences between WT and transgenic lines are indicated by asterisks (**P < 0.01; Student's *t*‐test). (e) Electrolyte leakage increment ratio represents the fold change of electrolyte leakage in transgenic lines relative to WT after cold treatment. A log_2_‐transformed value <0 indicates a smaller increase in electrolyte leakage in transgenic lines compared with WT. CT, control; LT, low temperature.

Due to the unavailability of *cbf3* mutants in birch, we performed complementation experiments using the *A. thaliana* cbf3 mutant to validate the functions of the *BpCBF3*s. The results are presented in Figure 3. Seeds of the Arabidopsis SAIL_244_D02 (*cbf3*) mutant were purchased, and overexpression vectors of *AtCBF3* and the five *BpCBF3*s were constructed for complementation assays. The results demonstrated that all *CBF3* homologs significantly rescued the cold‐induced lethality phenotype of the *cbf3* mutant. After 48 h of low‐temperature treatment, nearly all *cbf3* mutant plants died. In the WT plants, although some individuals survived, their growth was severely impaired. In contrast, only a very small number of individuals died in all complementation lines expressing *BpCBF3*s or *AtCBF3*. These findings suggest that the *BpCBF3*s function similarly to *AtCBF3* in conferring cold tolerance, and that functional redundancy exists among these homologous genes (Figure S5).

### Overexpression of *BpCBF3d* does not affect growth in birch

To validate whether *BpCBF3d* functions *in planta*, we performed functional analyses. Overexpression vectors for *BpCBF3*s driven by the *p35S* and *pRD29A* promoters were constructed. A total of 10 *BpCBF3* transgenic overexpression lines were generated via *Agrobacterium*‐mediated transformation of birch zygotic embryos. Plant height increment was evaluated for each transgenic line. The results showed that, regardless of the promoter used, the *BpCBF3e*, *BpCBF3d*, and *BpCBF3a* overexpression lines exhibited no obvious growth abnormalities, whereas both the *pRD29A::BpCBF3b* and *BpCBF3c* lines displayed developmental retardation. To further assess the stress tolerance of the transgenic lines, electrolyte leakage (EL) assays were performed. The results showed that, under both control and low‐temperature treatment conditions, all transgenic lines exhibited significantly lower ion leakage rates compared with the WT. Moreover, analysis of the incremental changes in electrical conductivity before and after low‐temperature treatment revealed that the conductivity increases in all overexpression lines were smaller than that in the WT, indicating that the WT plants lost more electrolytes following cold stress. These findings suggest that overexpression of *BpCBF3*s enhances cold tolerance in transgenic birch lines (Figure 3; Figure S6). To further evaluate the physiological responses of *BpCBF3* overexpression lines under cold stress, we measured the activities of catalase (CAT), peroxidase (POD), and superoxide dismutase (SOD) in the leaves of 1‐month‐old birch seedlings under control conditions and after 3 h of cold treatment at 4°C. The results demonstrated that the overexpression lines exhibited greater increases in enzyme activities upon cold exposure compared with the WT, which is consistent with the EL data. Collectively, these results further support the conclusion that overexpression of *BpCBF3*s confers enhanced low‐temperature tolerance in transgenic birch plants (Figure S7).

We further examined the expression levels of downstream *COR* genes in the transgenic lines under both normal growth conditions and low‐temperature treatment. The results showed that only the *BpCBF3d*‐overexpressing line exhibited comprehensive upregulation of all target genes (Figure S8). We subsequently analyzed the low‐temperature responses of downstream genes in the transgenic lines, focusing on *BpCOR47*, *BpKIN*, and *BpERD*, which are rapidly induced within 3 h. The expression patterns of these downstream genes under low‐temperature conditions were largely consistent with those observed under control conditions, albeit with moderately elevated amplitudes (Figure S9). Consistently, the *BpCBF3d*‐overexpressing line displayed the greatest increase in downstream gene expression. Collectively, these findings indicate that although functional redundancy exists among the *BpCBF3* members, *BpCBF3d* plays the most central role in gene regulation in birch.

### Heterologous expression of *AtRD29A* leads to its constitutive expression in birch

Notably, experimental observations revealed that the *AtRD29A* gene is constitutively expressed in birch, in contrast to its stress‐inducible expression pattern in Arabidopsis. In Arabidopsis, the stress‐inducible nature of *RD29A* has been extensively used to generate stress‐responsive transgenic lines. However, because all previously reported *RD29A* promoters originate from Arabidopsis, we hypothesized that *RD29A* might be specific to the Brassicaceae family. To test this hypothesis, we performed amino acid sequence alignments across all available plant genomes. *RD29A* homologs were detected exclusively in 29 Brassicaceae species. Further annotation of genes adjacent to *RD29A* on chromosomes revealed conserved synteny, where *RD29A* consistently neighbors its paralog *RD29B*, followed by *SHOC1* (transcribed in the opposite orientation) and a C2 NT TYPE PROTEIN gene (same transcriptional direction). The Brassicaceae family evolved relatively late, and no *RD29A* homologs were found in species closely related to Brassicaceae. Therefore, we proposed that *RD29A* originated within Brassicaceae during evolution. Although *Carica papaya* belongs to the same order (Brassicales), no *RD29A* homolog is mapped in its genome. Similarly, no *RD29A* homologs were found in woody or leguminous plants, although *RD29B*, *SHOC1*, and the C2 NT TYPE PROTEIN, located at adjacent positions on the same chromosome, could be mapped in these groups. In magnoliids, *RD29B*, *SHOC1*, and the C2 NT TYPE PROTEIN were mapped individually on separate chromosomes. We also conducted sequence alignments across representative Solanaceae and Poaceae species but did not detect any homologs of these four genes (Figure S10; Table S3).

**Figure 4:**
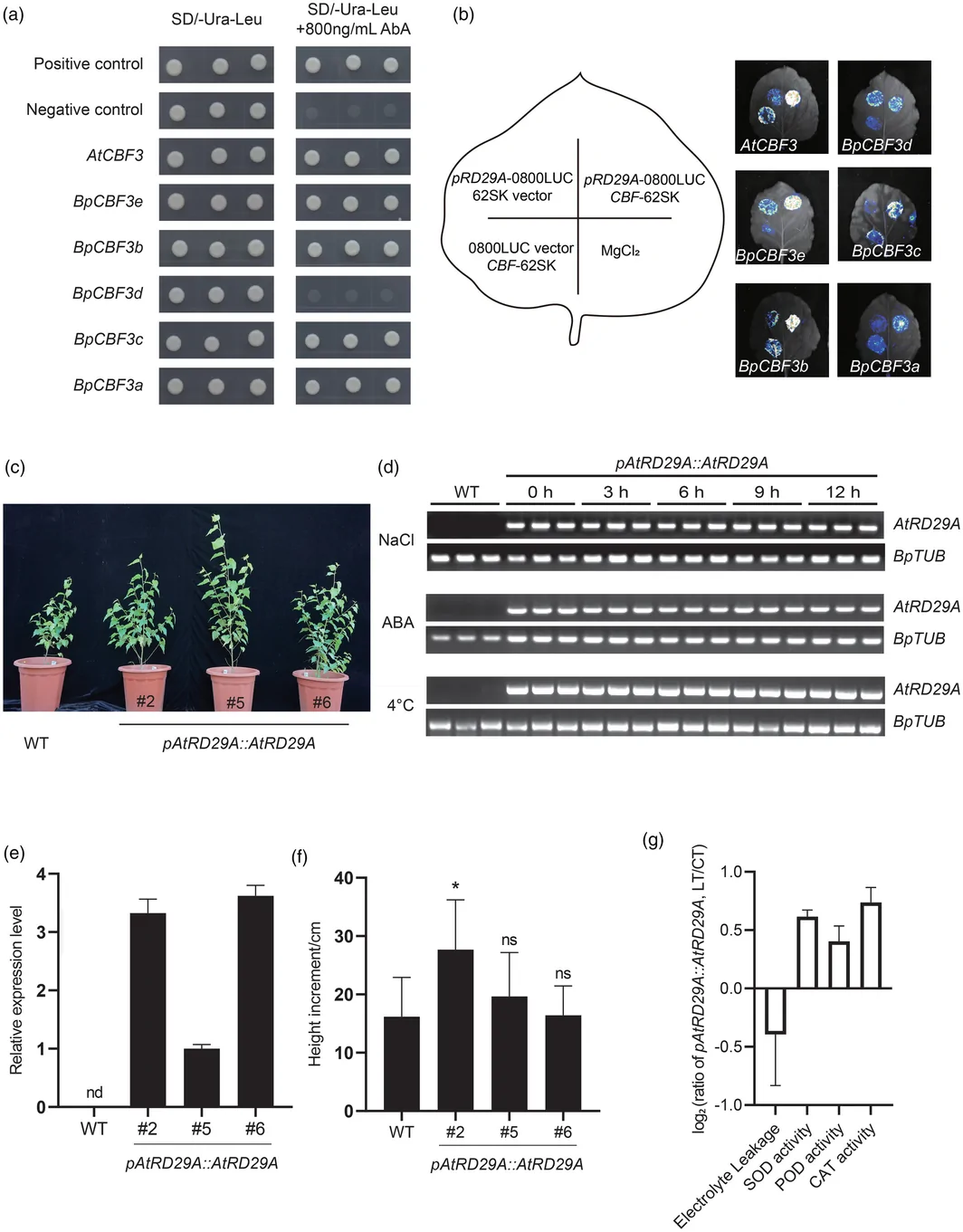
*AtRD29A* expressed constitutively in birch. (a) Yeast one‐hybrid assay of interactions between *AtCBF3*, *BpCBF3*s and the promoter of *AtRD29A*. Yeast strain was co‐transformed with the promoter of AtRD29A (as bait) cloned into the *pABAi* vector, and *AtCBF3* and *BpCBF3*s (as prey) cloned into the *pGADT7* vector. Interactions were indicated by growth on selection medium (SD/–Ura–Leu + 800 ng ml^−1^ AbA). Growth on SD/–Ura–Leu medium was used as a control. (b) Transcriptional activation activity assessed by a dual‐luciferase reporter assay using the *0800‐LUC* reporter and *62SK* effector vectors. Left panel: schematic diagram of the *Agrobacterium* infiltration sites on *Nicotiana benthamiana* leaves. Right panel: representative luminescence images showing luciferase (LUC) activity in infiltrated leaf discs. Enhanced luminescence intensity in the test sample relative to the negative control indicates transcriptional activation of the reporter gene by the tested effector. The assay was performed with at least three independent biological replicates, and representative results are shown. (c) Phenotype of *pRD29A::AtRD29A* plants compared with wild type (WT). Plants were grown in soil for 1 year. Bar = 20 cm. (d) Expression of *AtRD29A* in *pRD29A::AtRD29A* transgenic plants. RT‐PCR analysis was performed with 25 cycles. Each lane was loaded with 10 ng of total cDNA prepared from transgenic birch plants that had been treated with 100 mM NaCl, 100 μM ABA, or transferred to 4°C. The stress treatments were continued for 12 h and sampled every 3 h. Experiments were performed in triplicate. (e) Expression levels of the heterologous *AtRD29A* in transgenic birch lines (nd, not detected). (f) Height increment of heterologous transgenic birch lines. Plants were transferred to soil in September of the first year. Tree height was measured in August and September of the second year, and the increment was calculated as the difference between the two measurements. Asterisks and ‘ns’ indicate significant and non‐significant differences, respectively, compared with WT (*P < 0.05; Student's *t*‐test). (g) Log_2_‐transformed values of multiple physiological indicators, including electrolyte leakage, peroxidase (POD), superoxide dismutase (SOD), and catalase (CAT). A log_2_‐transformed value <0 indicates that the increase in electrolyte leakage of transgenic lines was smaller than that of WT after cold treatment. A log_2_‐transformed value >0 indicates that the increase in enzyme activity of transgenic lines was greater than that of WT after cold treatment.

The observation that *AtCBF3* induces *AtRD29A* expression in Arabidopsis, whereas *BpCBF3*s lack this regulatory capacity in birch, suggested a correlation between constitutive *AtRD29A* expression and the functional divergence of *BpCBF3* in birch, thereby prompting further investigation. Computational prediction indicated that *BpCBF3e*, *BpCBF3b*, *BpCBF3c*, and *BpCBF3a* can bind to the *AtRD29A* promoter, whereas *BpCBF3d* lacks detectable binding affinity (Figure S11). Y1H assays confirmed these predictions, displaying identical binding profiles (Figure 4a), which were further supported by transcriptional activation assays (Figure 4b). For *in planta* verification, we constructed a *pAtRD29A::AtRD29A* vector and heterologously expressed the full *AtRD29A* genomic sequence (−1282 to +2397 bp, including four exons and three introns) in birch (Figure 4c). After exposing the transgenic seedlings to multiple stress treatments, constitutive *AtRD29A* expression in birch was quantitatively validated (Figure 4d,e). Phenotypic analysis showed that *AtRD29A* transgenic lines exhibited significantly greater height increment compared to WT plants (Figure 4f). Consistent with the above findings, EL and enzyme activity assays revealed that heterologous *RD29A* expression significantly enhanced the stress tolerance of transgenic birch lines (Figure 4g; Figure S12).

## DISCUSSION

**Figure 5:**
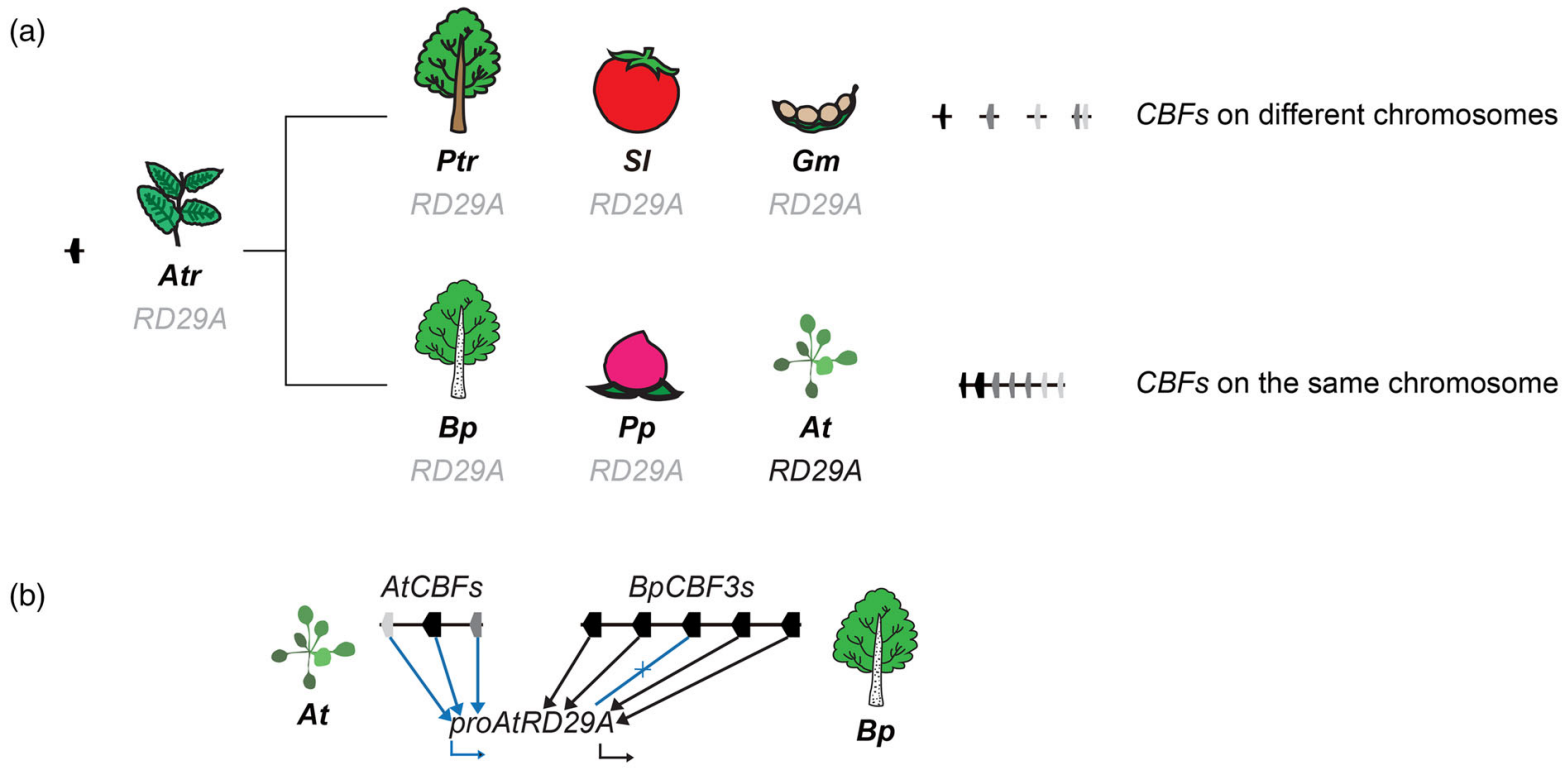
Evolutionary pattern of C‐repeat/DREB binding factors (*CBF*s). (a) *Amborella trichopoda* (Atr) contains only a single *CBF3* homologou. During subsequent evolution, *CBF*s diverged into two distinct evolutionary pathways: one where multiple *CBF* genes differentiated and became irregularly distributed across different chromosomes, as seen in species like poplar (Ptr), soybean (Gm), and tomato (Sl); and another where multiple CBF genes formed clustered tandem repeats on the same chromosome, such as in birch (Bp), peach (Pp), and Arabidopsis (At). Furthermore, among the examined species, the direct downstream gene *RD29A* of the *CBF* regulatory pathway was found exclusively in Brassicaceae. Different colors on the chromosome represent distinct *CBF* genes. Gray‐colored RD29A indicates the absence of this gene in the corresponding species, while black represents its presence. (b) The birch genome contains a total of five *CBF3*s. Among them, the *BpCBF3d* gene, although strongly induced by cold, does not bind to the *AtRD29A* promoter. The other four *BpCBF3* genes (*BpCBF3a*, *BpCBF3b*, *BpCBF3c*, *BpCBF3e*) can all bind to *AtRD29A* under normal conditions but exhibit a weak response to cold induction. Therefore, in birch, *AtRD29A* is predominantly bound by *BpCBF3e*, *BpCBF3b*, *BpCBF3c*, and *BpCBF3a* under normal conditions, resulting in a constitutively highly expressed pattern. Following cold stress, the expression levels of *BpCBF3e*, *BpCBF3b*, *BpCBF3c*, and *BpCBF3a* exhibit a modest increase, but this is insufficient to elevate *AtRD29A* expression. While *BpCBF3d* is strongly induced by cold, it cannot bind to the *AtRD29A* promoter region. Consequently, *AtRD29A* expression in birch is constitutive and exhibits virtually no change before and after cold stress treatment. The blue arrows indicate the gene can be cold‐induced.

According to our evolutionary analysis, the basal angiosperm *A. trichopoda* contains only a single *CBF3* homolog. During subsequent evolution, *CBF* genes diverged into two distinct evolutionary pathways: one in which multiple *CBF* genes diversified and became irregularly distributed across different chromosomes (for example, in poplar, soybean, and tomato), and another in which multiple *CBF* genes formed clustered tandem repeats on the same chromosome (for example, in peach and Arabidopsis). Birch represents a unique case, having undergone duplication to generate five tandemly repeated *CBF3* homologs on chromosome 11, although only *BpCBF3d* serves as the primary functional *CBF* gene. Furthermore, among all species analyzed, the downstream *CBF* target gene *RD29A* was detected exclusively in Brassicaceae (Figure 5a).

Collectively, these findings indicate that *BpCBF3d* in birch shares a high protein sequence similarity with *AtCBF3* in *A. thaliana* and possesses an identical EE‐Like motif in its promoter region. It displays diurnal rhythmicity, is induced by cold, and activates all downstream *COR* genes without impairing growth or development, thereby identifying it as the primary functional *CBF* ortholog in birch. However, birch lacks *RD29A* homologs, and further investigations confirmed that *RD29A* exists exclusively within Brassicaceae. Notably, heterologous expression of *AtRD29A* in birch converts its expression from inducible to constitutive. We performed bioinformatic prediction, *in vitro* validation, and *in vivo* verification to demonstrate that the constitutive expression of heterologous *RD29A* in birch is associated with *BpCBF3d*. This represents a critical functional divergence between *BpCBF3d* and *AtCBF3*: *BpCBF3d* does not bind the *RD29A* promoter. Therefore, we proposed divergent regulatory mechanisms for *AtRD29A* in birch and Arabidopsis. In Arabidopsis, *AtCBF3* is minimally expressed under normal conditions, insufficient to drive *RD29A*. Cold stress triggers rapid *AtCBF3* upregulation, inducing *RD29A* expression, followed by feedback‐mediated attenuation of both genes, resulting in stress‐induced *RD29A* expression. In birch, *BpCBF3d* is cold‐inducible, but cannot bind the *RD29A* promoter. Conversely, *BpCBF3a*, *BpCBF3b*, *BpCBF3c*, and *BpCBF3e* strongly bind *RD29A* but exhibit weak cold responsiveness, collectively leading to constitutive *RD29A* expression (Figure 5b).

We noticed that in 2022, Nie et al. identified 17 *BpCBF*s in *Betula pendula* (Nie et al., 2022), and in 2023, Zhang et al. identified 20 *BpCBF*s in *Betula platyphylla* (Zhang et al., 2023). In contrast, we initially identified only 12 *BpCBF*s in our birch material (Figure S1). To investigate the discrepancy in the number of *CBF* genes among different birch accessions, we conducted a detailed analysis. We downloaded the respective reference genomes from the corresponding studies and performed a cross‐comparison of the *CBF* genes identified in those studies with the reference genome used in our study. Among the 17 *BpCBF*s identified in *B. pendula*, 13 genes were distributed as two gene clusters on two chromosomes, while the remaining four were singletons (Bpev01.c2812.g0001, Bpev01.c2812.g0002, Bpev01.c0478.g0011, and Bpev01.c0884.g0001). Among these four singletons, Bpev01.c2812.g0001 and Bpev01.c2812.g0002 were located on the same contig, which was subsequently discarded during chromosome integration in later genome version updates. Bpev01.c0478.g0011 and Bpev01.c0884.g0001 had no homologous counterparts in either Zhang's or our reference genome. In *B. platyphylla*, among the 20 *BpCBF*s identified by Zhang et al., 19 genes formed two gene clusters on two chromosomes, with one additional singleton (BPChr10G03390) located on another chromosome. Similarly, this singleton showed no homology in Nie's or our reference genome. Across the three birch reference genomes, the initially identified *CBF* genes consistently formed two gene clusters. Sequence alignment revealed high conservation of seven genes within one cluster across all three genomes. However, based on transcriptomic data and cold stress responses reported by Zhang and Nie, none of these seven genes were cold‐inducible. In contrast, all cold‐responsive *CBF*s were concentrated in the other cluster. Within this cold‐responsive cluster, our study as well as that of Nie et al. in *B. pendula* consistently identified only five cold‐responsive *CBF* genes, that is, the *BpCBF3*s that are the focus of this study. In contrast, Zhang et al. identified six cold‐responsive *CBF* genes in this region in *B. platyphylla*. Further analysis revealed that, due to differences in genome assembly, the genomic region corresponding to the *BpCBF3e* gene in our study was annotated as an intron in our reference genome, whereas Zhang's reference genome considered this region as protein‐coding. To verify the actual structure of this region in the *B. platyphylla* genome, we designed primers flanking the BpCBF3e gene and performed PCR amplification using cDNA from *B. platyphylla* as a template. The resulting amplicon was approximately 600 bp, and sequencing alignment confirmed that it was consistent with the annotation of our reference genome (Figure S13).

Birch, a fast‐growing and globally distributed tree species, survives harsh high‐latitude winters through its inherently high cold tolerance (Wang et al., 2023, 2025). Given this pre‐existing adaptation, the transgenic overexpression lines did not exhibit a further increase in cold resistance. In the present study, we attempted to construct CRISPR/Cas9 vectors targeting five *CBF3* genes in birch. However, despite multiple attempts, no knockout mutants were obtained. This failure could be attributed to the essential role of *CBF3* genes in birch survival, where their deletion presumably causes lethality, whereas the high sequence homology among these five genes further highlights their functional importance.

Phenotypic observations of 1‐year‐old transgenic birch seedlings revealed distinct growth patterns among the five transgenic lines (Figure 4; Figure S5). Regardless of promoter type, the transgenic lines did not exhibit a uniform growth phenotype. *BpCBF3e* driven by *p35S* displayed growth comparable to the WT controls but exceeded WT growth when expressed under the pRD29A promoter. Transgenic lines expressing *BpCBF3b* under *p35S* displayed marginally enhanced growth compared to WT, yet showed significantly reduced growth when driven by *pRD29A*. Both *BpCBF3d* and *BpCBF3a* maintained growth levels indistinguishable from that of WT under both promoter systems, whereas *BpCBF3c* consistently exhibited reduced growth in both *p35S*‐ and *pRD29A*‐driven lines relative to WT. This phenotypic variation appeared to be independent of *CBF* expression levels—mirroring documented differential growth phenotypes in *A. thaliana* overexpressing *DREB1A* (Kasuga et al., 1999). A similar phenomenon has been observed in *A. thaliana* transgenic lines overexpressing *CBF3*, which exhibit both growth‐retarded and normal growth phenotypes (Kasuga et al., 1999). The growth retardation phenotypes observed in *CBF3* transgenic lines primarily result from gibberellin deficiency and can be restored by exogenous gibberellin application (Cong et al., 2008). Alternatively, the diverse growth phenotypes observed among *CBF/DREB1* overexpression lines may be related to functional diversification within the *CBF* family during evolution. Multiple *DREB1* genes in rice are phylogenetically more distantly related to those in *A. thaliana*, suggesting functional divergence (Deng et al., 2024). Moreover, overexpression of *OsDREB1C* induces early flowering and increased yield in transgenic lines, further demonstrating functional divergence within the *CBF/DREB1* gene family across species (Wei et al., 2022).

The promoter region of *AtRD29A* contains multiple stress‐responsive cis‐elements, establishing its role as a key marker gene in stress‐resistance research. This further suggests that the constitutive expression of *AtRD29A* in birch is unlikely to result solely from *BpCBF3*s activity and that birch may lack critical repressor genes required to suppress *RD29A* expression. The combination of these factors leads to exceptionally high levels of heterologously expressed *AtRD29A* in birch. Following the generation of *RD29A* genomic transgenic lines, phenotypic analysis revealed that the transgenic birch exhibited significantly enhanced growth compared to WT plants. These observations indicate that heterologous *AtRD29A* expression may improve adaptive fitness in birch.

## MATERIALS AND METHODS

### Plant materials and growth conditions

Birch plants were maintained at the State Key Laboratory of Tree Genetics and Breeding, Northeast Forestry University (Harbin, Heilongjiang Province, China). Plantlets were cultured in sterile plastic bottles containing 100 ml culture media (WPM supplemented with 0.8 mg L^−1^ 6‐BA, 0.02 mg L^−1^ NAA, and 20 g L^−1^ sucrose). After 2 months of tissue culture, the plantlets were transferred to a rooting medium (WPM supplemented with 0.2 mg L^−1^ NAA and 20 g L^−1^ sucrose) and subsequently transplanted into soil for 1 month of additional growth. All plants were grown in a greenhouse under an average temperature of 25°C, 60% relative humidity, and a 16‐h light:8‐h dark photoperiod before being transferred outside.

### Identification of *CBF* members in birch

Six CBF protein sequences from *A. thaliana* (AT4G25490, AT4G25470, AT4G25480, AT5G51990, AT1G12610, AT1G63030) were used as templates to construct an HMMER model. The HMMER 3.1b2 software was employed to search the birch genome for CBF/DREB1 genes with an *E*‐value threshold of <1 × 10^−10^. The birch genome used in this study is openly available in the Genome Sequence Archive at the National Genomics Data Center (https://ngdc.cncb.ac.cn/gsa/) under accession number PRJCA040696 (Chen et al., 2025). Based on phylogenetic analysis and protein sequence similarity, five *CBF3* genes were ultimately identified in birch.

### Phylogenetic analysis

To validate the function of *CBF* genes in plants, we selected 20 species for phylogenetic tree analysis. Only angiosperms were included for this analysis because *CBF/DREB1* proteins have not been detected in algae, mosses, ferns, or gymnosperms (Li, Chen, et al., 2020). The 20 selected species represented diverse lineages, including evolutionarily primitive magnoliopsida: Amborella (*A. trichopoda*), Colorado blue columbine (*A. coerulea*), California poppy (*E. californica*), and Resurrection plant (*M. flabellifolia*); Poaceae monocots: rice (*Oryza sativa*), maize (*Zea mays*), and barley (*Hordeum vulgare*); crops from Leguminosae and Solanaceae: soybean (*G. max*), barrel medic (*M. truncatula*), common bean (*P. vulgaris*), tomato (*S. lycopersicum*), and potato (*S. tuberosum*); woody plants from Rosaceae and Salicaceae: apple (*M. domestica*), peach (*P. persica*), poplar (*P. trichocarpa*), and willow (*S. purpurea*); birch (*B. platyphylla*) from Betulaceae; as well as Brassicaceae species: field mustard (*Brassica rapa*), pink shepherd's‐purse (*Capsella rubella*), and the model plant *A. thaliana* (Figure S2).

MEGA X software was used to construct phylogenetic trees for the *CBF* gene families across multiple species.

### Subcellular localization assay

For subcellular localization analysis, the coding sequences (CDSs) of *AtCBF3* and five *BpCBF3* homologs (*BpCBF3e*, *BpCBF3b*, *BpCBF3d*, *BpCBF3c*, and *BpCBF3a*) were individually cloned into the binary vector pCAMBIA1300, which harbors a green fluorescent protein (GFP) tag. The resulting recombinant constructs were transformed into *Agrobacterium tumefaciens* strain GV3101. The agrobacterial cultures were grown to an optical density of OD_600_ = 0.8, then harvested and resuspended in infiltration buffer. Subsequently, the suspensions were infiltrated into the abaxial airspaces of fully expanded leaves of 4‐ to 6‐week‐old *Nicotiana benthamiana* plants using a needleless syringe. After infiltration, the plants were grown under normal conditions for 48 h. GFP fluorescence signals were then visualized and captured using a fluorescence microscope. The subcellular localization of the fusion proteins was determined based on the distribution pattern of GFP fluorescence.

### Complementation assay in Arabidopsis

The Arabidopsis mutant line SAIL_244_D02, obtained from the ABRC (formerly Arashare) stock center, was used for complementation assays. All plants were grown in Percival growth chambers under a 16 h light/8 h dark photoperiod.

For complementation, the CDSs of *AtCBF3* and five *BpCBF3* homologs (*BpCBF3e*, *BpCBF3b*, *BpCBF3d*, *BpCBF3c*, and *BpCBF3a*) were individually cloned into the binary vector pCAMBIA1300. The resulting constructs were introduced into *A. tumefaciens* strain EHA105 and transformed into the SAIL_244_D02 mutant plants via the floral dip method.

Transgenic T1 seeds were harvested and surface‐sterilized. The seeds were then germinated and selected on half‐strength Murashige and Skoog (1/2 MS) medium supplemented with BASTA and kanamycin. After 10 days of growth on the selection medium, the surviving seedlings were subjected to cold treatment at 0°C for 48 h. Following the cold stress, the seedlings were returned to normal growth conditions for a 5‐day recovery period. The survival rate was then recorded as the percentage of green, viable seedlings relative to the total number of treated seedlings.

### Screening and identification of transgenic plants

WT birch RNA was used as a template to clone the five *BpCBF3* genes. WT *A. thaliana* (Col) DNA was used as a template to clone *AtRD29A*. pCAMBIA3301 was used as the *p35S* carrier, and the promoter element in pCAMBIA3301 was replaced with the *RD29A* promoter. Homologous recombination between the vector and target genes was performed following double enzyme digestion. The transgene was introduced into birch zygotic embryos via transformation, and positive lines were identified by PCR and DNA sequencing.

### Electrolyte leakage assays

EL was used to evaluate freezing tolerance and was measured according to (Zhao, Zhang, et al., 2016). Birch leaves of uniform size were collected and placed into 15 ml tubes containing 10 ml of deionized water. The control group was kept at room temperature, while the treatment group was exposed to −20°C for 1 h. After the treatment, the leaves from both the control and treatment groups, along with the solution in each tube, were transferred together into 50 ml tubes containing 25 ml of deionized water. The tubes were gently shaken overnight, and the initial conductivity was measured and recorded as S1. The tubes were then autoclaved at 121°C for 20 min. After autoclaving, the solution was shaken for an additional 3 h, and the final conductivity was measured as S2. EL was calculated as follows: Electrolyte leakage (%) = (S1/S2) × 100. Three biological replicates were performed for each treatment.

To quantitatively evaluate the magnitude of change in EL of the transgenic lines relative to the WT following low‐temperature treatment, we calculated the log_2_‐transformed ratio as follows:

$${\text{log}}_{2}‐\text{transformed ratio}={\text{log}}_{2}\frac{\mathrm{EL}\_\text{overexpression}\_\mathrm{LT}/\mathrm{EL}\_\mathrm{WT}\_\mathrm{LT}}{\mathrm{EL}\_\text{overexpression}\_\mathrm{CT}/\mathrm{EL}\_\mathrm{WT}\_\mathrm{CT}}$$

where LT denotes low‐temperature treatment and CT represents control conditions. A ratio greater than 0 indicates that the transgenic lines exhibited a greater change in EL upon cold treatment compared with the WT, whereas a ratio less than 0 indicates a smaller change relative to the WT.

### Measurement of antioxidant enzyme activities

Leaves from 1‐month‐old birch rooted seedlings grown under normal control conditions or subjected to low‐temperature stress (4°C for 3 h) were collected for antioxidant enzyme assays. For each sample, 0.1 g of fresh leaf tissue was quickly transferred to a pre‐chilled 1.5 ml centrifuge tube and ground into a fine powder in liquid nitrogen. Subsequently, 1.0 ml of pre‐cooled extraction buffer (supplied by Solarbio Science & Technology Co., Ltd., Beijing, China) was added, and the mixture was thoroughly vortexed and homogenized on ice. The homogenate was then centrifuged at 8000× ***g*** for 10 min at 4°C. The resulting supernatant was collected as the crude enzyme extract and kept on ice for further analysis.

The activities of CAT, POD, and SOD were determined using the corresponding assay kits (Solarbio, Beijing, China) strictly following the manufacturer's instructions. The absorbance values of each sample were measured at the specified wavelengths. Enzyme activities were calculated according to the formulas provided in the respective kit manuals. All physiological measurements were conducted with three independent biological replicates to ensure data reliability.

To quantitatively evaluate the magnitude of change in enzyme activities of the transgenic lines relative to the WT following low‐temperature treatment, we calculated the log_2_‐transformed ratio as follows:

$$\;{\text{log}}_{2}‐\text{transformed ratio}={\text{log}}_{2}\frac{\text{enzyme}\_\text{overexpression}\_\mathrm{LT}/\text{enzyme}\_\mathrm{WT}\_\mathrm{LT}}{\text{enzyme}\_\text{overexpression}\_\mathrm{CT}/\text{enzyme}\_\mathrm{WT}\_\mathrm{CT}}$$

where LT denotes low‐temperature treatment and CT represents control conditions. A ratio greater than 0 indicates that the transgenic lines exhibited a greater change in enzyme activities upon cold treatment compared with the WT, whereas a ratio less than 0 indicates a smaller change relative to the WT.

### Prediction of binding sites

The binding sites of BpCBF3s in the *AtRD29A* promoter were predicted using the Alphafold3 online platform, and the results were visualized using PyMOL software.

### Yeast one‐hybrid assay

To test the interaction between CBF proteins and the *AtRD29A* promoter, the CDS of each *BpCBF3* gene was cloned into the prey vector pGADT7. The promoter fragment of *AtRD29A* was cloned into the bait vector pAbAi. The pAbAi reporter construct was linearized by restriction enzyme digestion and integrated into the genome of yeast strain Y1HGold. The pGADT7 prey constructs were then transformed into the bait‐containing yeast competent cells. Transformants were selected on SD/–Leu medium. Positive colonies were suspended in 0.9% NaCl and plated onto SD/–Leu medium containing AbA (Aureobasidin A) to assess protein–DNA interactions. Yeast growth on AbA‐containing medium indicates a positive interaction between the CBF protein and the *AtRD29A* promoter.

### Transcriptional activation assay

Transcriptional activation activity was assessed using a *N. benthamiana*‐based dual‐luciferase (LUC) reporter assay. The CDSs of *AtCBF3* and five *BpCBF3* homologs (*BpCBF3a*, *BpCBF3b*, *BpCBF3c*, *BpCBF3d*, and *BpCBF3e*) were individually cloned into the effector vector 62SK. The reporter vector 0800LUC carried the firefly LUC gene driven by the *RD29A* promoter. All constructs were introduced into *A. tumefaciens* strain GV3101. The agrobacterial cultures were grown to an optical density of OD_600_ = 1.0, then harvested and resuspended in infiltration buffer. The reporter and effector constructs were mixed at a ratio of 1:10 (0800LUC:62SK) prior to infiltration. The mixed suspensions were infiltrated into the fully expanded leaves of 4‐ to 6‐week‐old *N. benthamiana* plants using a needleless syringe. After infiltration, the plants were first kept in darkness for 24 h, followed by 48 h of culture under normal growth conditions. LUC activities were then visualized and recorded using an *in vivo* plant imaging system.

## CONFLICT OF INTEREST

The authors have not declared a conflict of interest.

## DECLARATION OF AI USE

During the preparation of this revised manuscript, the authors used DeepSeek to assist with language refinement and grammar correction. The tool was used solely for improving the clarity and readability of the text. All AI‐generated content was critically reviewed and edited by the authors, who assume full responsibility for the accuracy and integrity of the final manuscript.

## Supporting information


**Figure S1.** Identification of *CBF*s in birch.
**Figure S2.** Evolutionary timeline of 20 plant species.
**Figure S3.** Evolution of *DREB2* homologs.
**Figure S4.** Subcellular localization of CBF proteins.
**Figure S5.** Complementation analysis of the Arabidopsis *cbf3* mutant.
**Figure S6.** Phenotypic characterization of *BpCBF3e*, *BpCBF3b*, *BpCBF3c* and *BpCBF3a* transgenic lines.
**Figure S7.** Enzyme activities of wild type and transgenic lines under control and low‐temperature conditions.
**Figure S8.** Expression levels of downstream genes in transgenic lines.
**Figure S9.** Expression levels of downstream genes in response to low‐temperature treatment.
**Figure S10.** The *RD29A* gene is found exclusively in Brassicaceae.
**Figure S11.** Online prediction results.
**Figure S12.** Physiological responses of *RD29A* heterologous transgenic lines under low‐temperature stress.
**Figure S13.** Comparative analysis of *CBF* copy number variation among different birch species.

**Table S1.** *CBF*s in 20 plant species.

**Table S2.** Rename of *CBF*s in 20 plant species.

**Table S3.** *RD29A* homologous genes and adjacent genes.

## Data Availability

The data presented in this study are openly available in the Genome Sequence Archive (GSA) at https://ngdc.cncb.ac.cn/gsa/ under GSA dataset accession numbers CRA027422 and CRA027602, with BioProject accession number PRJCA040696.
