# Supplementary material for: Constitutive AtRD29A expression reveals divergent BpCBF3 regulatory mechanisms in birch
Source: Plant J. 2026 Sep 25;127(6):e71121. doi: 10.1111/tpj.71121 (PMC13615328; doi:10.1111/tpj.71121)
Supplement: Supplementary file 1 [file TPJ-127-0-s004.pdf]

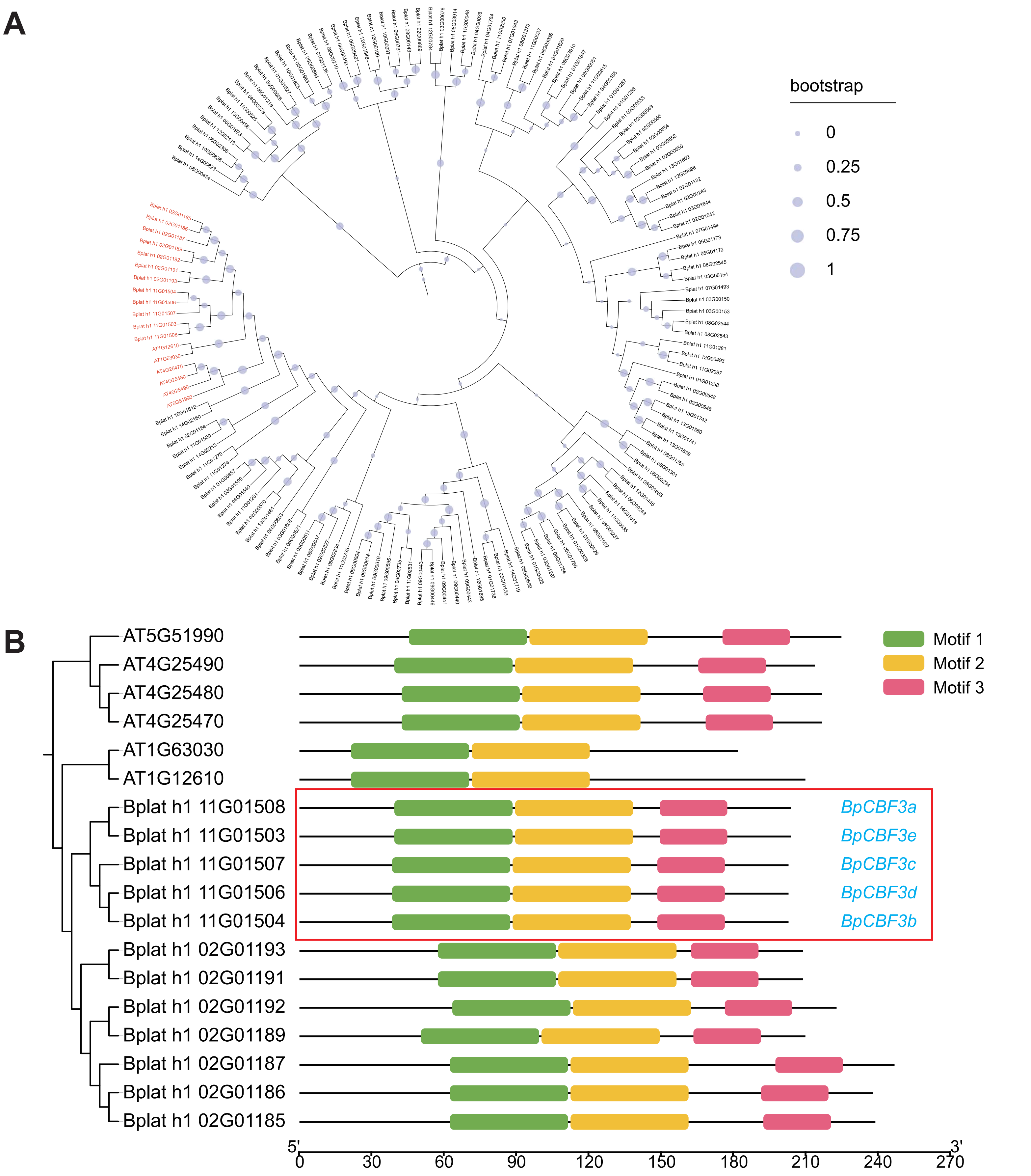

Figure S1 Identification of *CBF*s in birch.

A. Using the HMMER model, 145 *AP2* family genes were identified in birch. Phylogenetic analysis revealed 12 genes that are more closely related to the *CBF*s of *Arabidopsis thaliana* (highlighted in red in the figure).

B. MOTIF analysis was performed on six *Arabidopsis thaliana CBF* genes and 12 candidate *CBF* genes from birch. The results showed that five genes, arranged in tandem repeats on chromosome 11, share consistent motifs with *Arabidopsis thaliana* at the same positions (indicated by the red box in the figure). Based on their protein sequence similarity to the *CBFs* of *Arabidopsis thaliana*, these five genes were named *BpCBF3e, BpCBF3b, BpCBF3d, BpCBF3c*, and *BpCBF3a*, respectively.

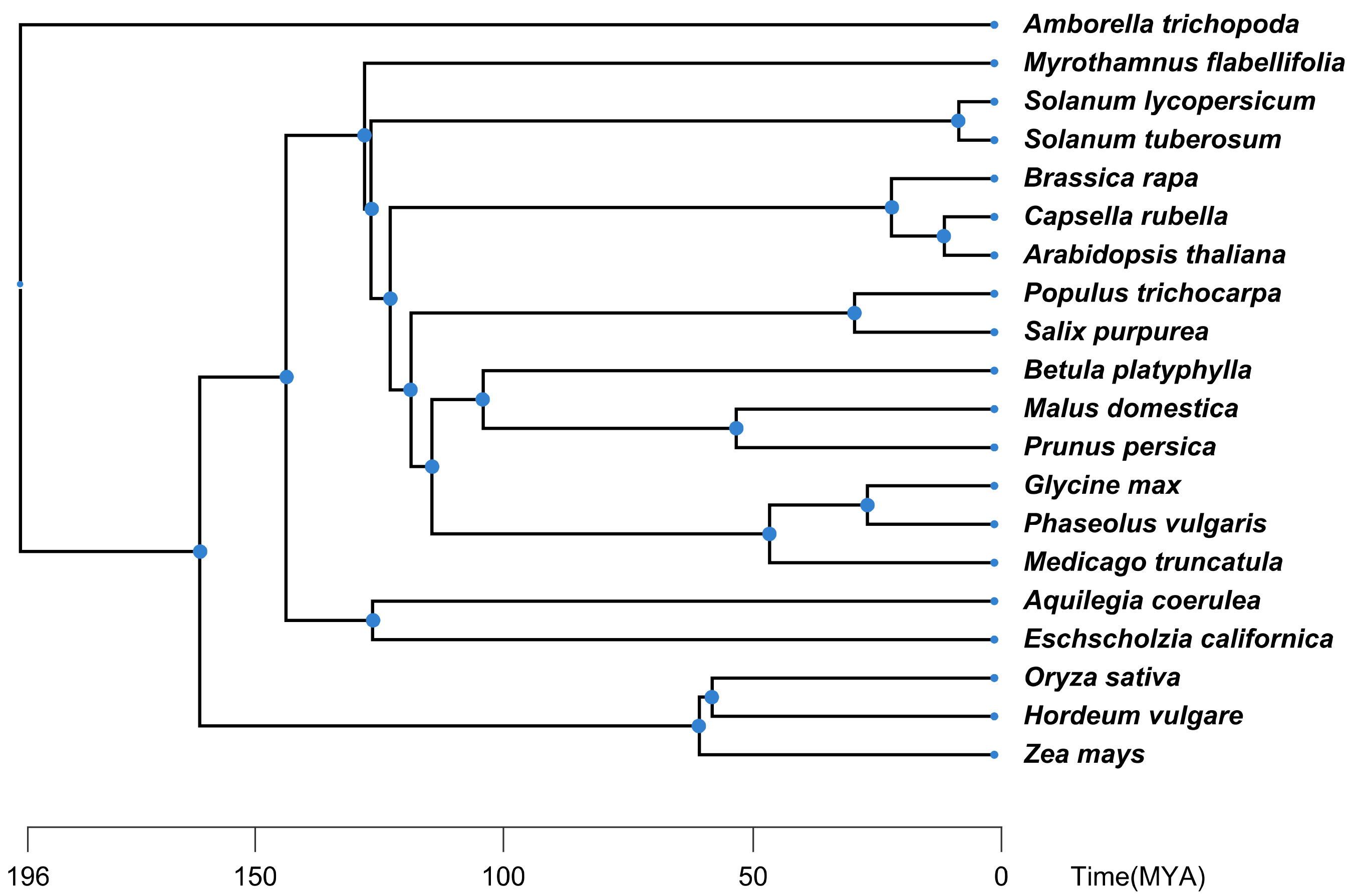

Figure S2 Evolutionary Timeline of 20 Plant Species.
The evolutionary timeline includes magnoliids, Poaceae monocots, Fabaceae, Solanaceae, woody plants, and Brassicaceae. The evolutionary timeline indicates that among these 20 species, the most ancient are the magnoliids, followed sequentially by Poaceae monocots, birch and rosaceous woody plants, Fabaceae and salicaceous woody plants, Brassicaceae, and Solanaceae. The horizontal axis at the bottom represents evolutionary time in MYA (Million Years Ago).

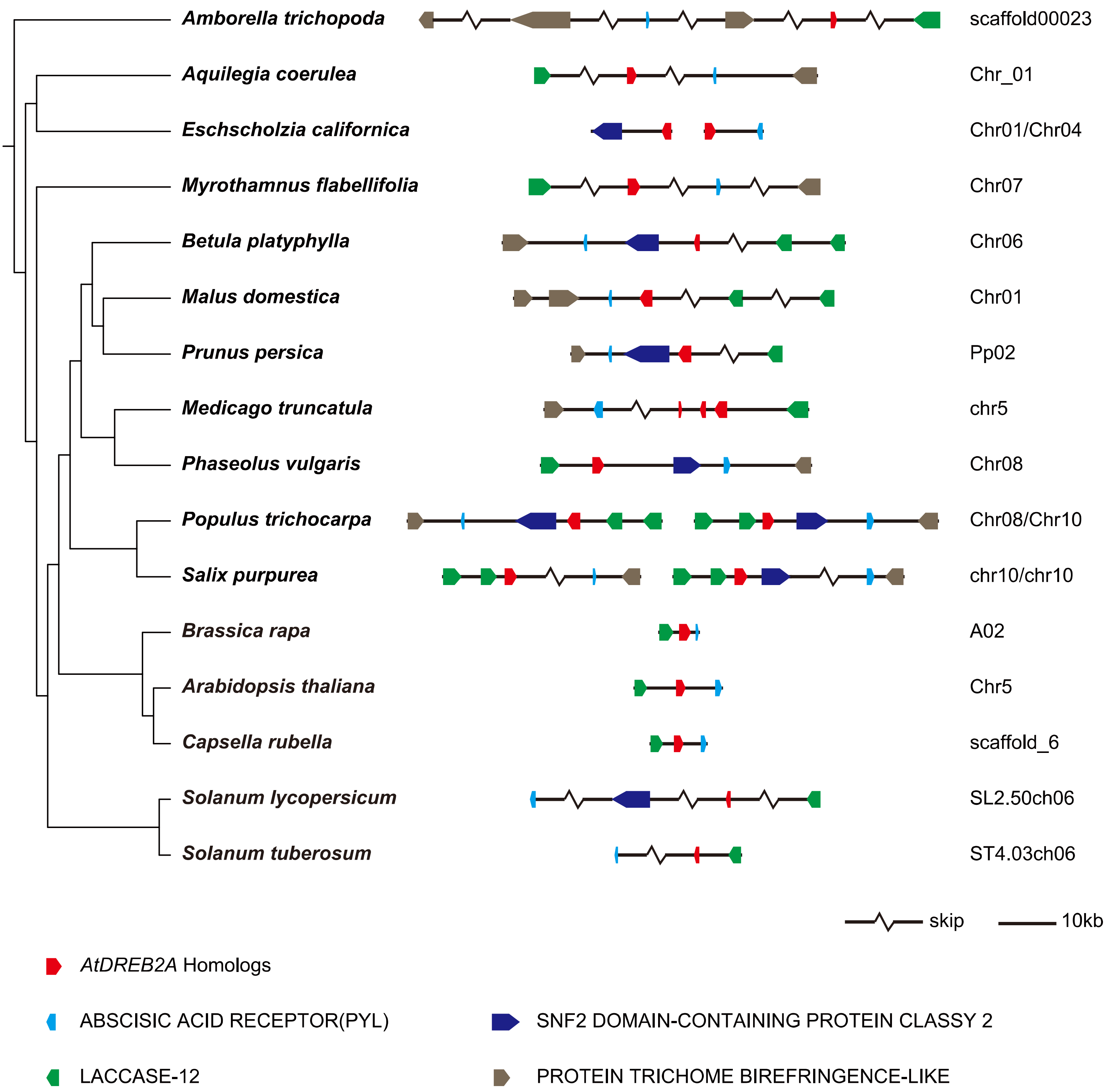

Figure S3 Evolution of *DREB2* Homologs

On the left side of the figure is the evolutionary timeline of the species included in the alignment. The right side shows the *DREB2* homologous genes identified through amino acid sequence alignment (genes highlighted in red), along with adjacent genes on the same chromosome (genes in other colors). The direction of the arrows indicates the transcription orientation of the genes, and genes of the same color represent those annotated as homologous genes.

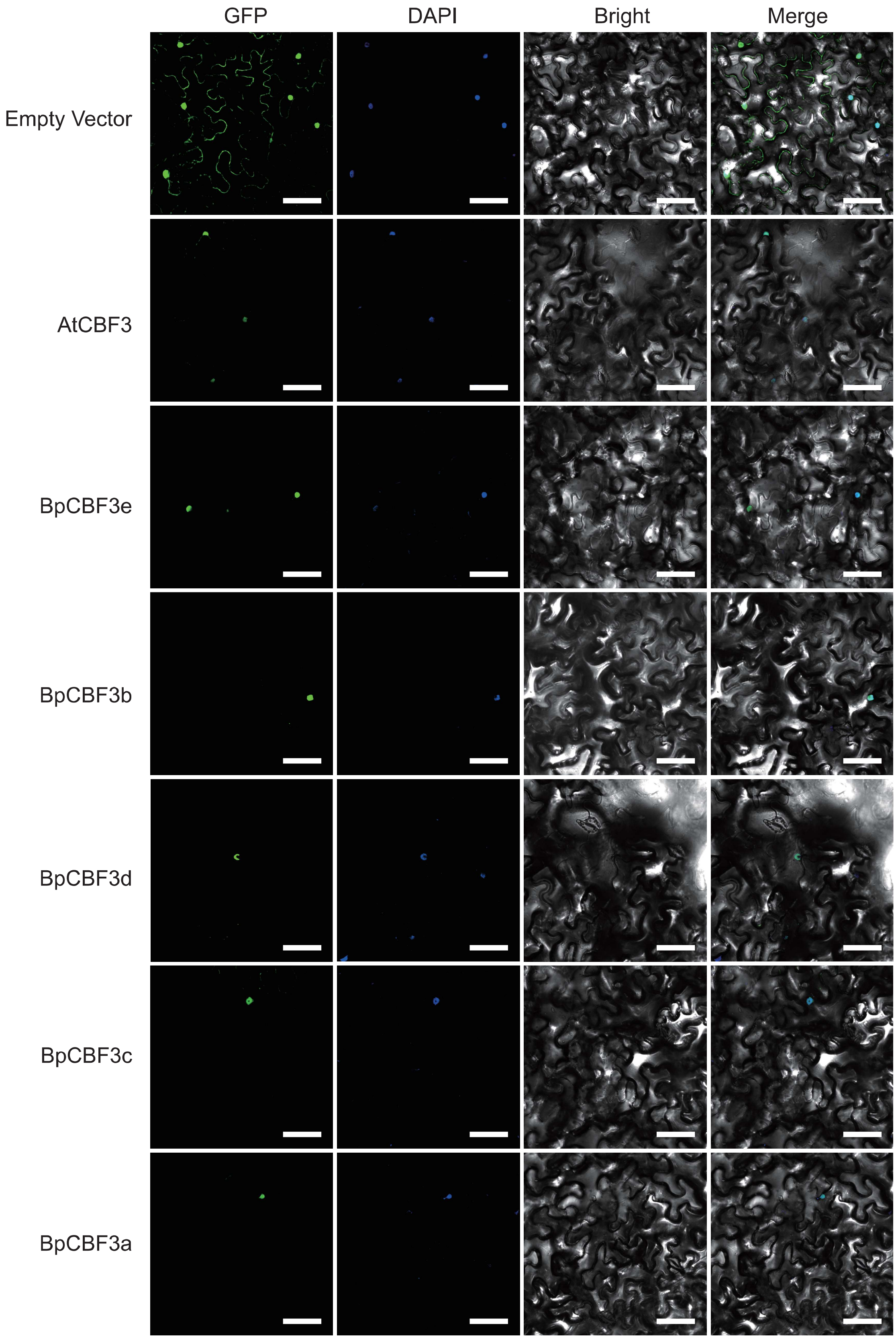

Figure S4 Subcellular localization of CBF proteins.
GFP-tagged CBF fusion proteins were transiently expressed in Nicotiana benthamiana leaves. DAPI was used for nuclear co-localization analysis. The empty vector served as a negative control, and AtCBF3 served as a positive control. From left to right: GFP fluorescence, DAPI staining, bright-field, and merged images. Scale bar = 50 μm.

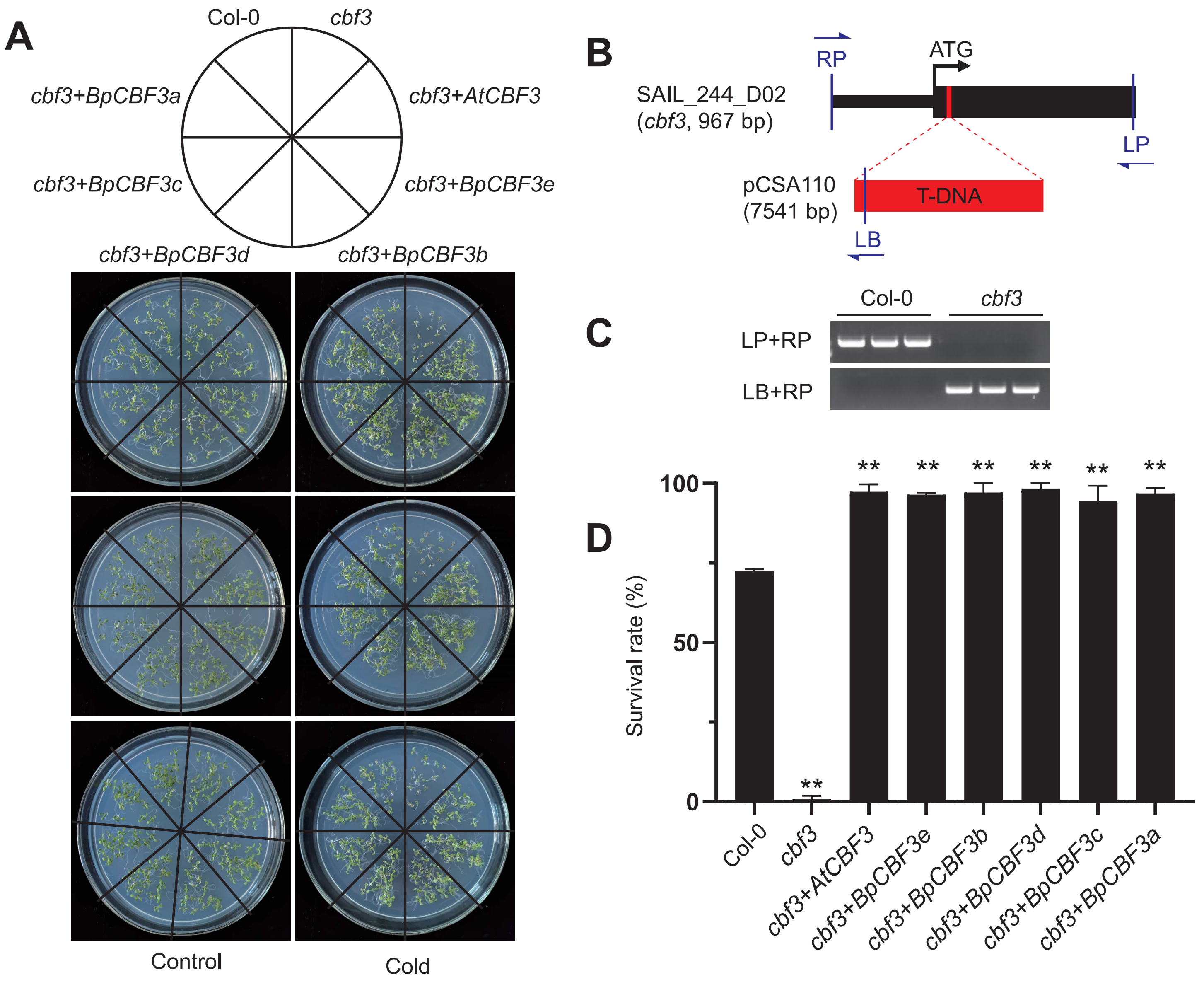

Figure S5 Complementation analysis of the *Arabidopsis cbf3* mutant.

A. Phenotypes of the *Arabidopsis* complementation lines under cold stress. Plates were incubated at 22 °C (16-h light/8-h dark). On day 7 after stratification, the cold-treated group was subjected to 0 °C for 48 h, while the control group was maintained under normal conditions (22 °C). Three independent replicates, representing three independent transgenic events carrying the same construct, are shown; in each horizontal pair of plates, the same seed types (genotypes) were placed at identical positions. The top schematic indicates the seed types at different positions on the plates.

B. Schematic representation of the *Arabidopsis cbf3* mutant locus, showing a T-DNA insertion (pCSA110) within the gene.

C. PCR-based genotyping using the three-primer method, confirming the T-DNA insertion in the cbf3 mutant lines.

D. Statistical analysis of survival rates after low-temperature treatment and subsequent recovery. Data represent the means ± SD from three independent biological replicates per line. Significant differences between each complementation line and the wild-type (WT) control were determined by Student's t-test. Asterisks indicate statistically significant differences (*P < 0.05, **P < 0.01).

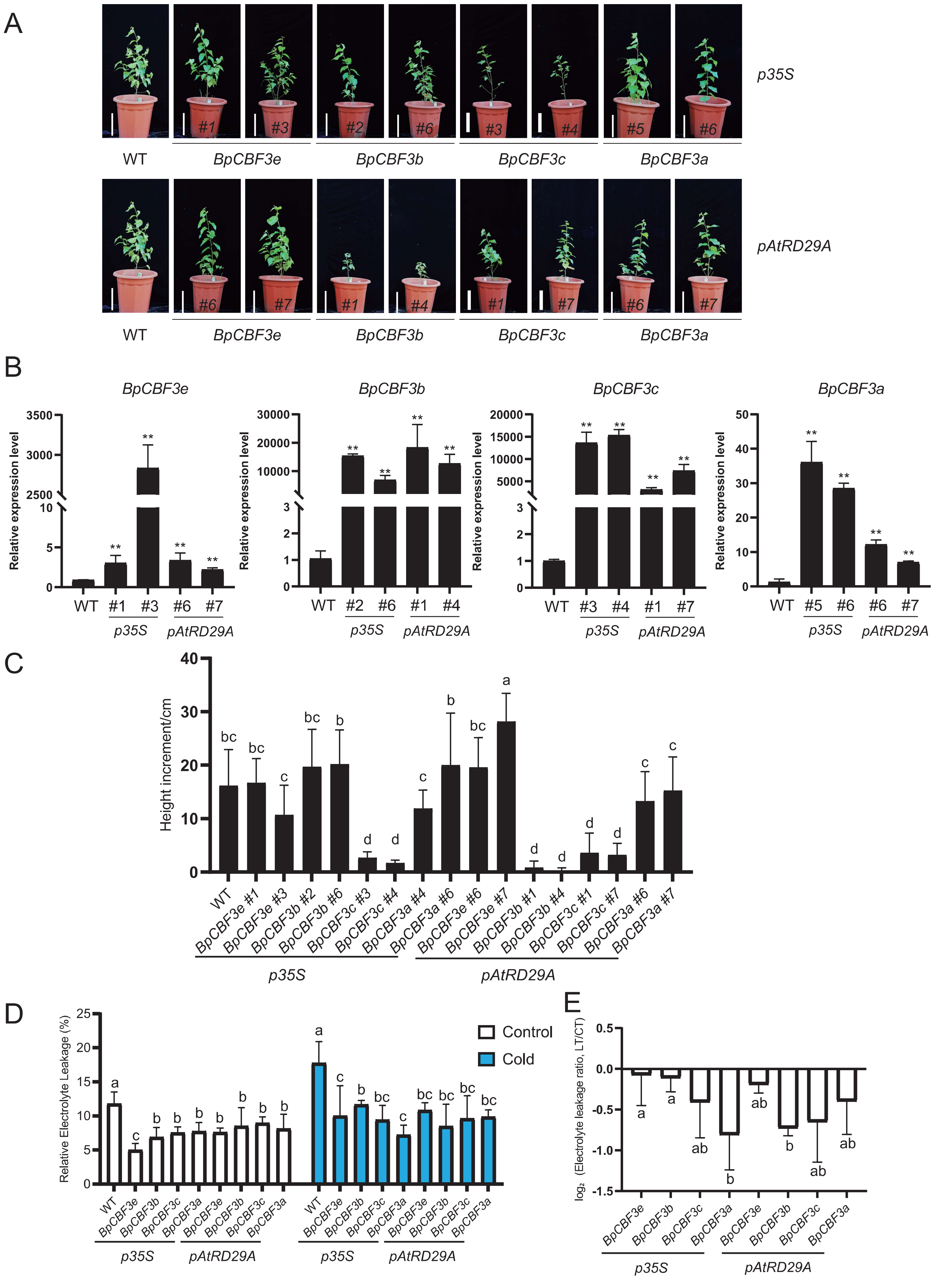
A
p35S
pAtRD29A
WT
BpCBF3e
BpCBF3b
BpCBF3c
BpCBF3a
#1 #3 #2 #6 #3 #4 #5 #6
#6 #7 #1 #4 #1 #7 #6 #7
B
BpCBF3e
BpCBF3b
BpCBF3c
BpCBF3a
Relative expression level
WT #1 #3 #6 #7
WT #2 #6 #1 #4
WT #3 #4 #1 #7
WT #5 #6 #6 #7
p35S
pAtRD29A
C
Height increment/cm
WT
BpCBF3e #1
BpCBF3e #3
BpCBF3b #2
BpCBF3b #6
BpCBF3c #3
BpCBF3c #4
BpCBF3a #4
BpCBF3a #6
BpCBF3e #6
BpCBF3e #7
BpCBF3b #1
BpCBF3b #4
BpCBF3c #1
BpCBF3c #7
BpCBF3a #6
BpCBF3a #7
p35S
pAtRD29A
D
Relative Electrolyte Leakage (%)
Control
Cold
E
log₂ (Electrolyte leakage ratio, LT/CT)

Figure S6 Phenotypic characterization of *BpCBF3e, BpCBF3b,BpCBF3c* and *BpCBF3a* transgenic lines.

A. Phenotypes of the *BpCBF3e, BpCBF3b*, *BpCBF3c* and *BpCBF3a* driven by the *p35S* and *pAtRD29A* promoters in relation to WT. Plants grown in soil for one year. Bar=20cm.

B. Relative gene expression levels of *BpCBF3e, BpCBF3b*, *BpCBF3c* and *BpCBF3a* driven by *p35S* and *pAtRD29A* in the field. Transcript levels were determined by qRT-PCR. Error bars represent mean ± SD from three biological replicates. Asterisks indicate significant differences (*$P < 0.05$, **$P < 0.01$; Student's t-test) between transgenic lines and WT.

C. Height increment of *BpCBF3e, BpCBF3b*, *BpCBF3c* and *BpCBF3a* driven by *p35S* and *pAtRD29A* in the field. Plants were transferred to soil in September of the first year. Tree height was measured in August and September of the second year, and the increment was calculated as the difference between the two measurements. Data are shown as mean ± SD (n = 3 biological replicates). Different letters indicate significant differences among genotypes under the same treatment condition ($P < 0.05$; Fisher's LSD test).

D. Electrolyte leakage of WT, *BpCBF3e, BpCBF3b*, *BpCBF3c* and *BpCBF3a* overexpression lines under control and cold treatment conditions. Electrolyte leakage was calculated as (S1/S2) × 100, where S1 and S2 represent the conductivity before and after autoclaving, respectively. Data are shown as mean ± SD (n = 3 biological replicates). Different letters indicate significant differences among genotypes under the same treatment condition ($P < 0.05$; Fisher's LSD test).

E. Electrolyte leakage increment ratio represents the fold change of electrolyte leakage in transgenic lines relative to WT after cold treatment. A $\log_2$-transformed value < 0 indicates a smaller increase in electrolyte leakage in transgenic lines compared with WT. CT, control; LT, low temperature.Data are shown as mean ± SD (n = 3 biological replicates). Different letters indicate significant differences among genotypes under the same treatment condition ($P < 0.05$; Fisher's LSD test).

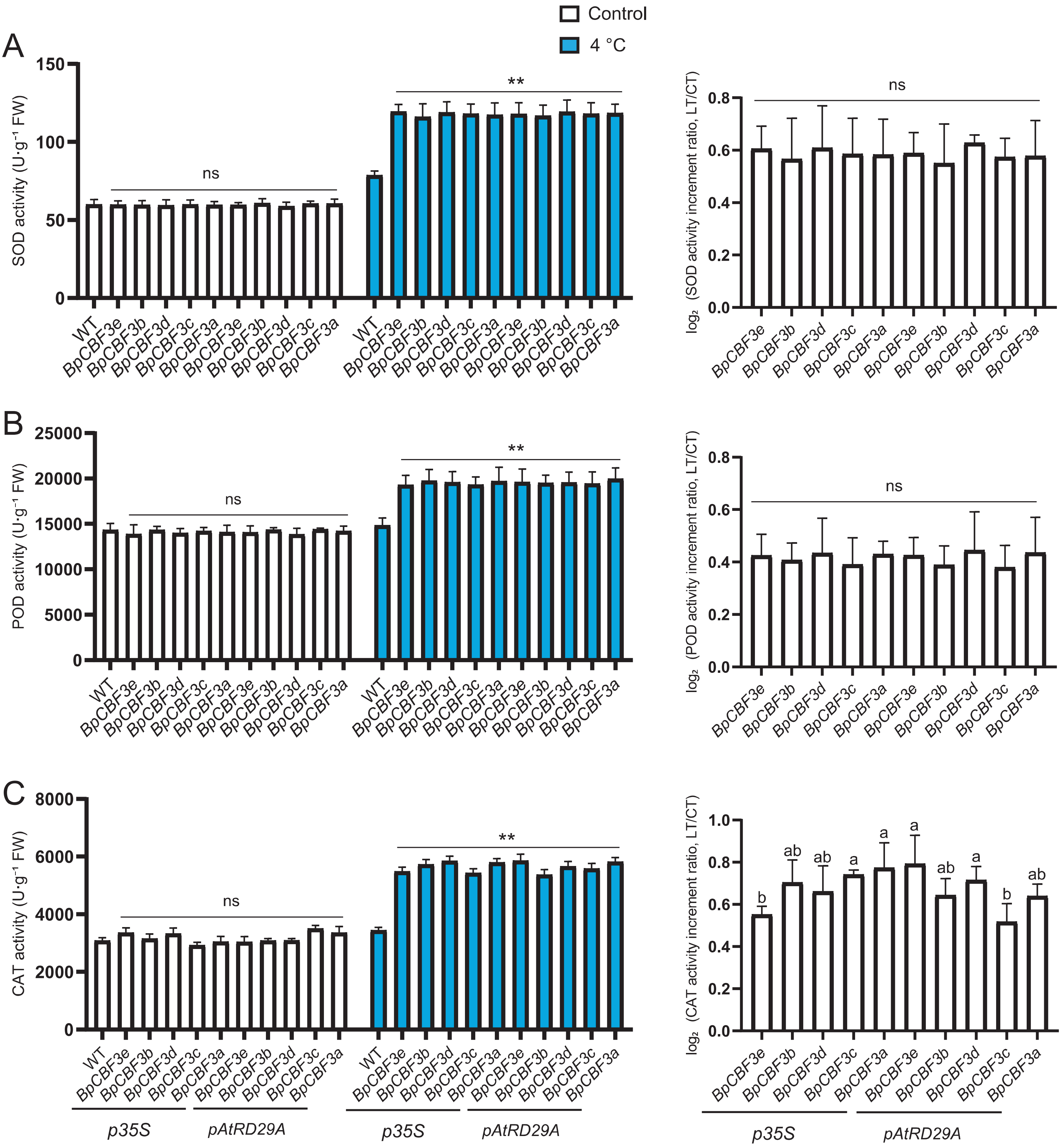

Figure S7 Enzyme activities of wild-type and transgenic lines under control and low-temperature conditions. Activities of POD, SOD, and CAT were measured in wild-type (WT) and transgenic lines under normal (control) and low-temperature treatment conditions. Data are presented as means ± SD. Statistical significance was determined by Student's t-test, and "ns" indicates no significant difference. Enzyme activity increment ratio represents the relative change in enzyme activities (POD, SOD, and CAT) of transgenic lines versus wild-type lines under low-temperature treatment. A log_2-transformed value > 0 indicates a larger increase in transgenic lines than in the wild type.

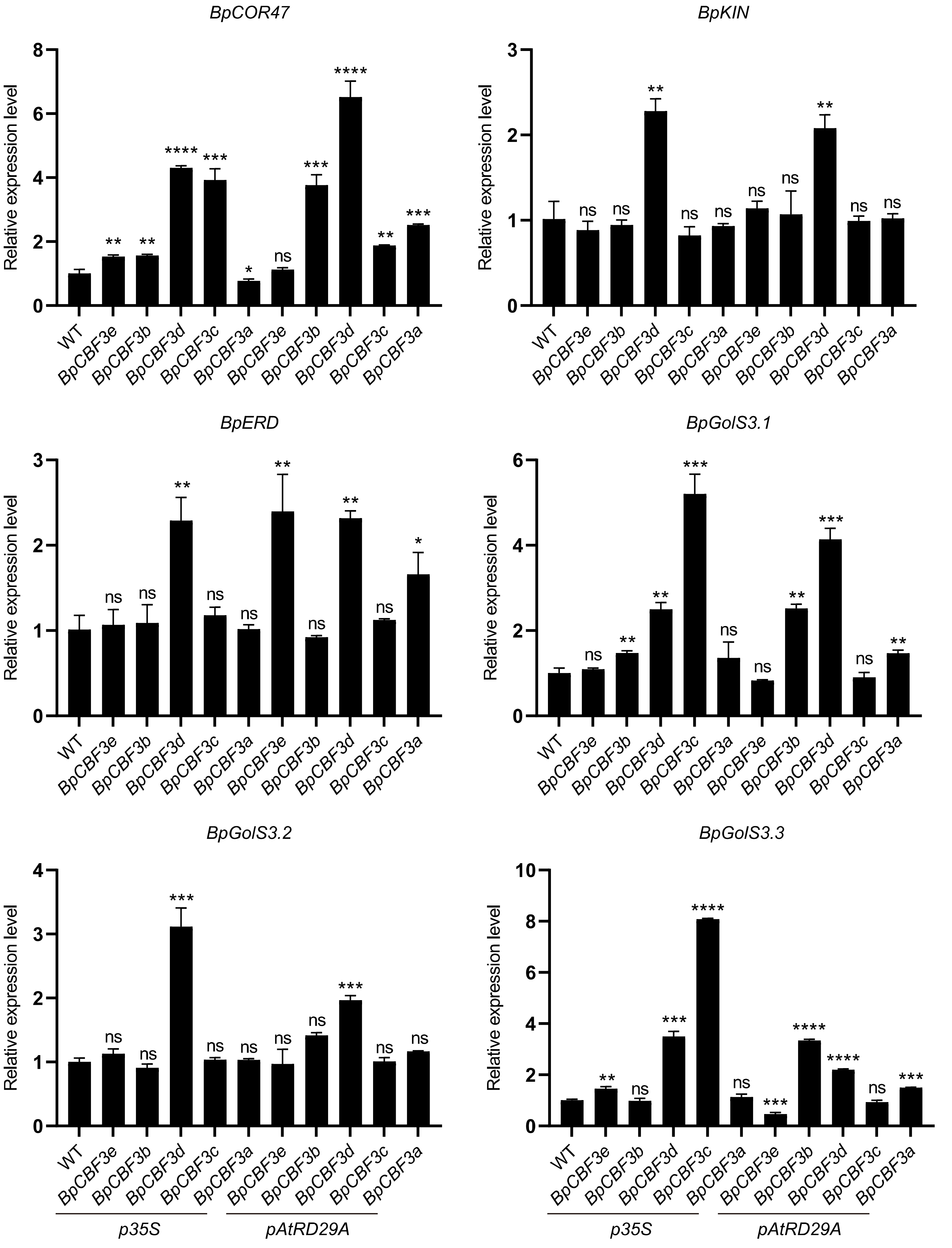

Figure S8 Expression levels of downstream genes in transgenic lines.

Expression levels of six downstream *COR* genes in transgenic lines.The experiment was repeated three times.The transcript levels of each genes were determined by qRT-PCR. The error bars refer to means ±SDs, experiment was repeated three times. The asterisks indicate significant differences (*P<0.05, **P<0.01 according to Student's t test) in the expression of each gene in the transgenic plants compared with that of the WT.

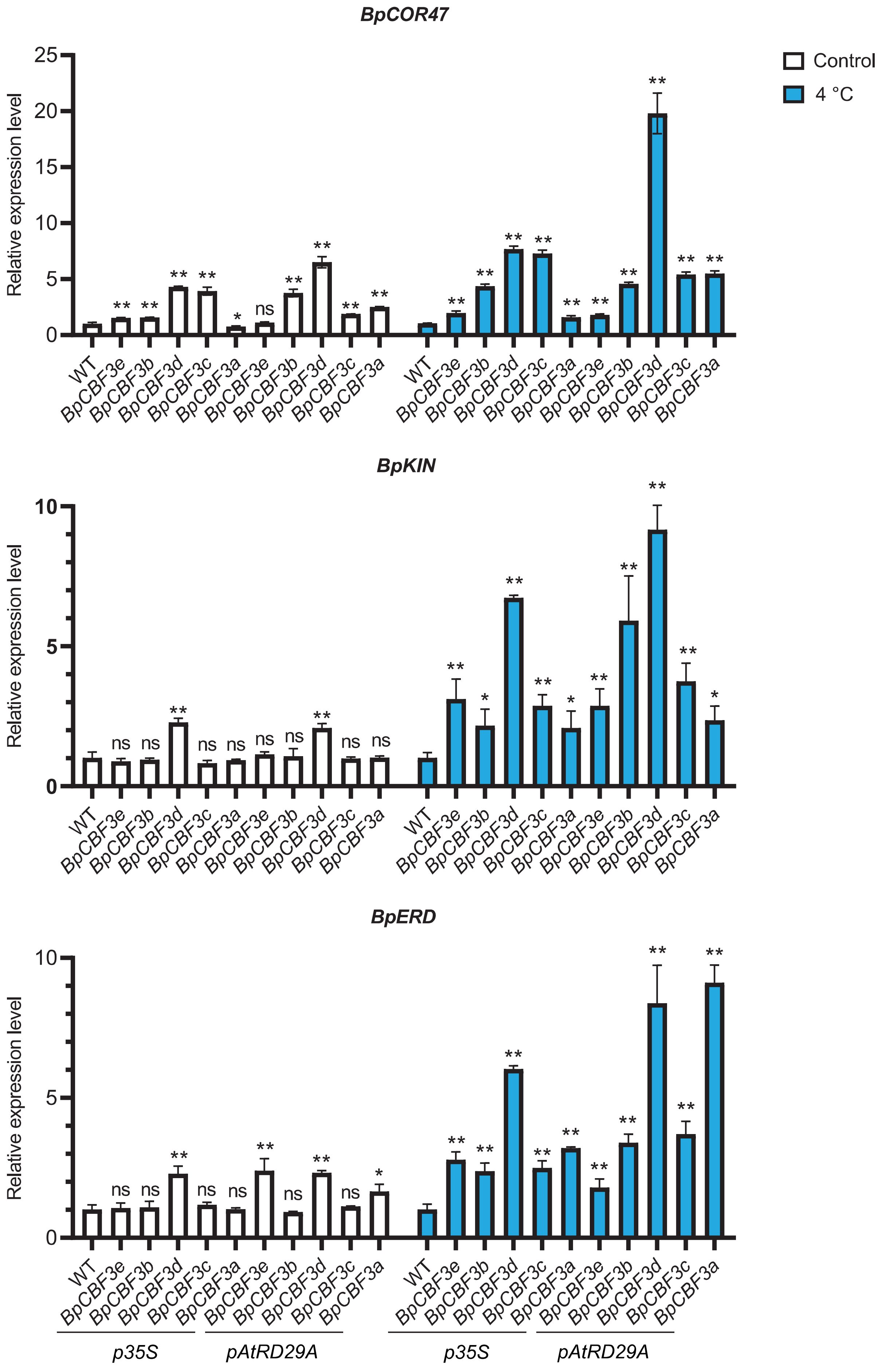

Figure S9 Expression levels of downstream genes in response to low-temperature treatment.
Transgenic lines were subjected to 4°C treatment, and the expression levels of the downstream genes *BpCOR47, BpKIN* and *BpERD* were examined after treatment.. The error bars refer to means ±SDs, experiment was repeated three times. The asterisks indicate significant differences (*P < 0.05, **P < 0.01 according to Student's t-test) in the expression of each gene in the transgenic plants compared with that of the WT. "ns" indicates no significant difference.

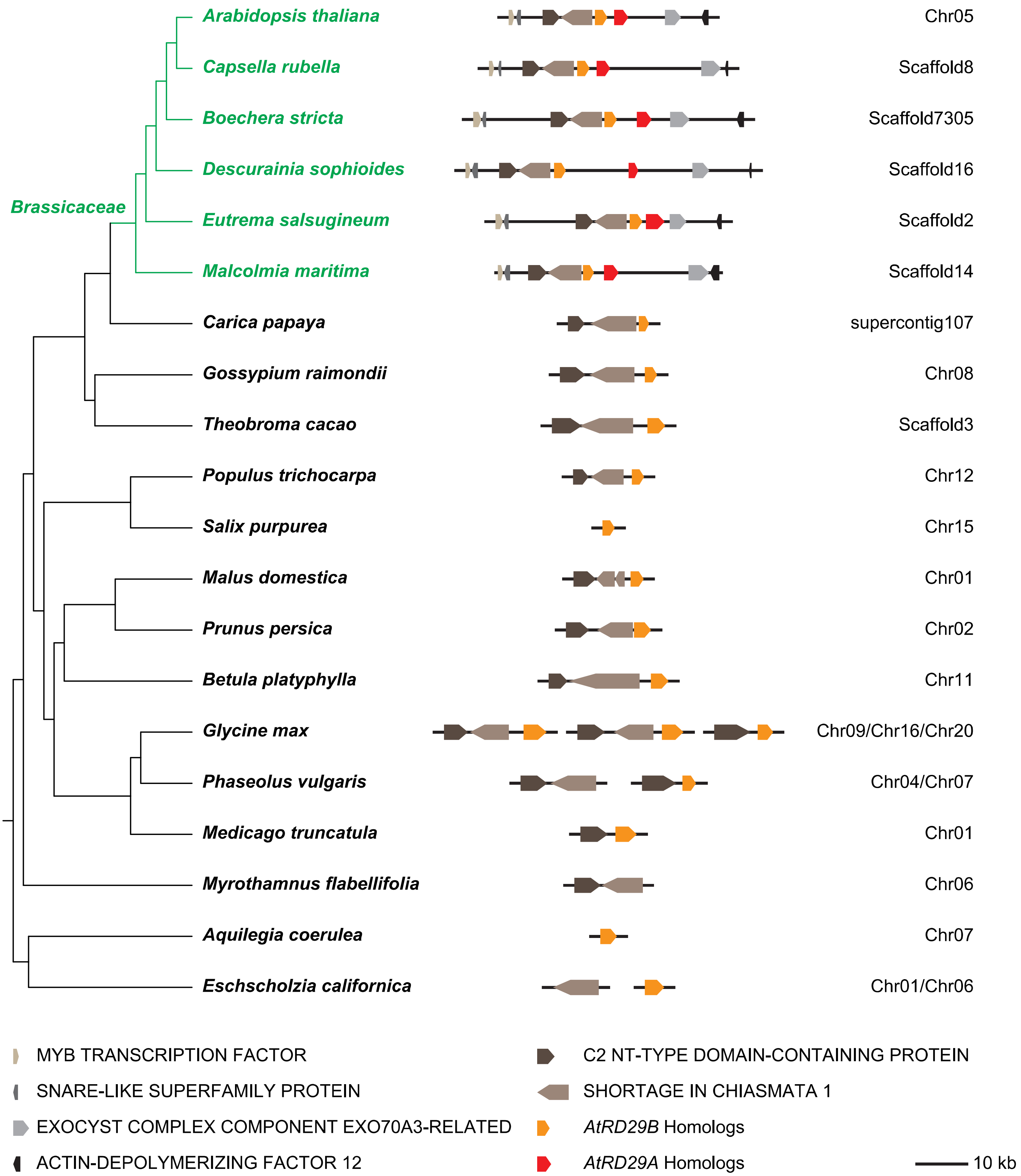

Figure S10 The *RD29A* gene is found exclusively in Brassicaceae.
On the left side of the figure is the evolutionary timeline of the species included in the alignment. Six representative species of Brassicaceae are highlighted in green. The right side shows the *AtRD29A* homologous genes identified through amino acid sequence alignment (genes highlighted in red), along with adjacent genes on the same chromosome (genes in other colors). In non-Brassicaceae species, where no *RD29A* gene was aligned, *RD29B* or *SHOC1* genes were identified via sequence alignment, ultimately confirming the absence of the *RD29A* gene in these other species on the chromosome. The direction of the arrows indicates the transcription orientation of the genes, and genes of the same color represent those annotated as homologous genes.

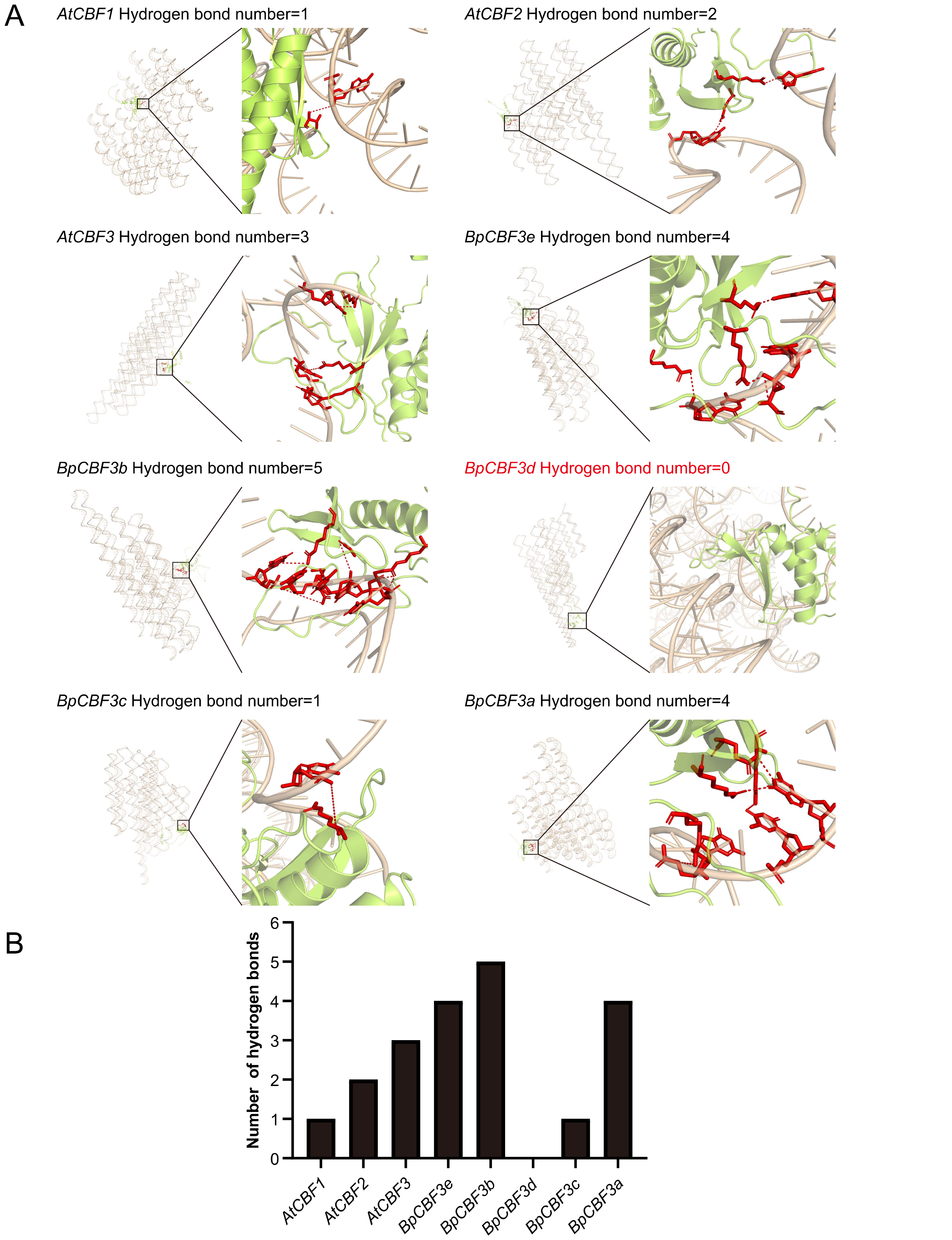

Figure S11 Online Prediction Results.

A. Schematic diagrams of hydrogen bond sites between the *AtRD29A* promoter and three *Arabidopsis CBF*s (*CBF1, CBF2, CBF3*) as well as five birch *CBF3*s (*BpCBF3a, BpCBF3b, BpCBF3c, BpCBF3d, BpCBF3e*), as predicted by AlphaFold3. Hydrogen bonds are indicated by dashed lines.

B. Statistical comparison of the number of hydrogen bonds formed in each protein–DNA complex. A higher number of hydrogen bonds indicates stronger binding affinity between the transcription factor and the *AtRD29A* promoter.

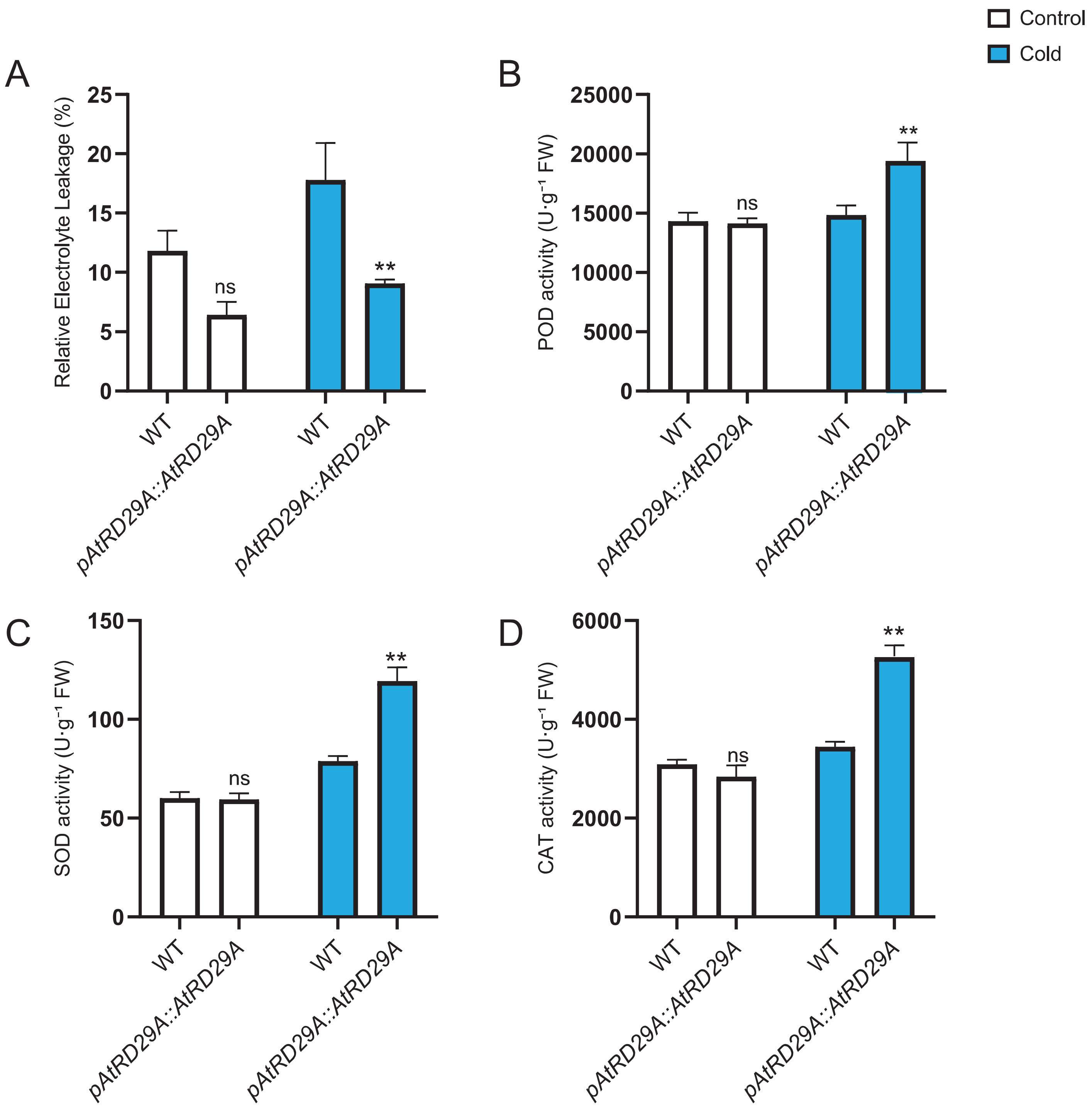

Figure S12 Physiological responses of *RD29A* heterologous transgenic lines under low-temperature stress. Electrolyte leakage and the activities of POD, SOD, and CAT were measured in wild-type (WT) and *RD29A* heterologous transgenic lines under normal (control) and low-temperature treatment conditions. Data are presented as means ± SD. Statistical significance was determined by Student's t-test, and "ns" indicates no significant difference. Asterisks indicate significant differences between transgenic lines and WT under the same condition (*P < 0.05, **P < 0.01).

	Numbers of putative *CBFs*	cluster 1	cluster 2 (cold-induced)	Solitary gene 1	Solitary gene 2	Solitary gene 3	Solitary gene 4	Solitary gene 5
Betula pendula (Nie et al. 2022)	17	7	6	Bpev01.c2812.g0001	Bpev01.c2812.g0002	Bpev01.c0478.g0011	Bpev01.c0884.g0001	-
Betula platyphylla (Zhang et al. 2023)	20	7	12	-	-	-	-	BPChr10G03390
Betula platyphylla (Our study)	12	7	5	-	-	-	-	-

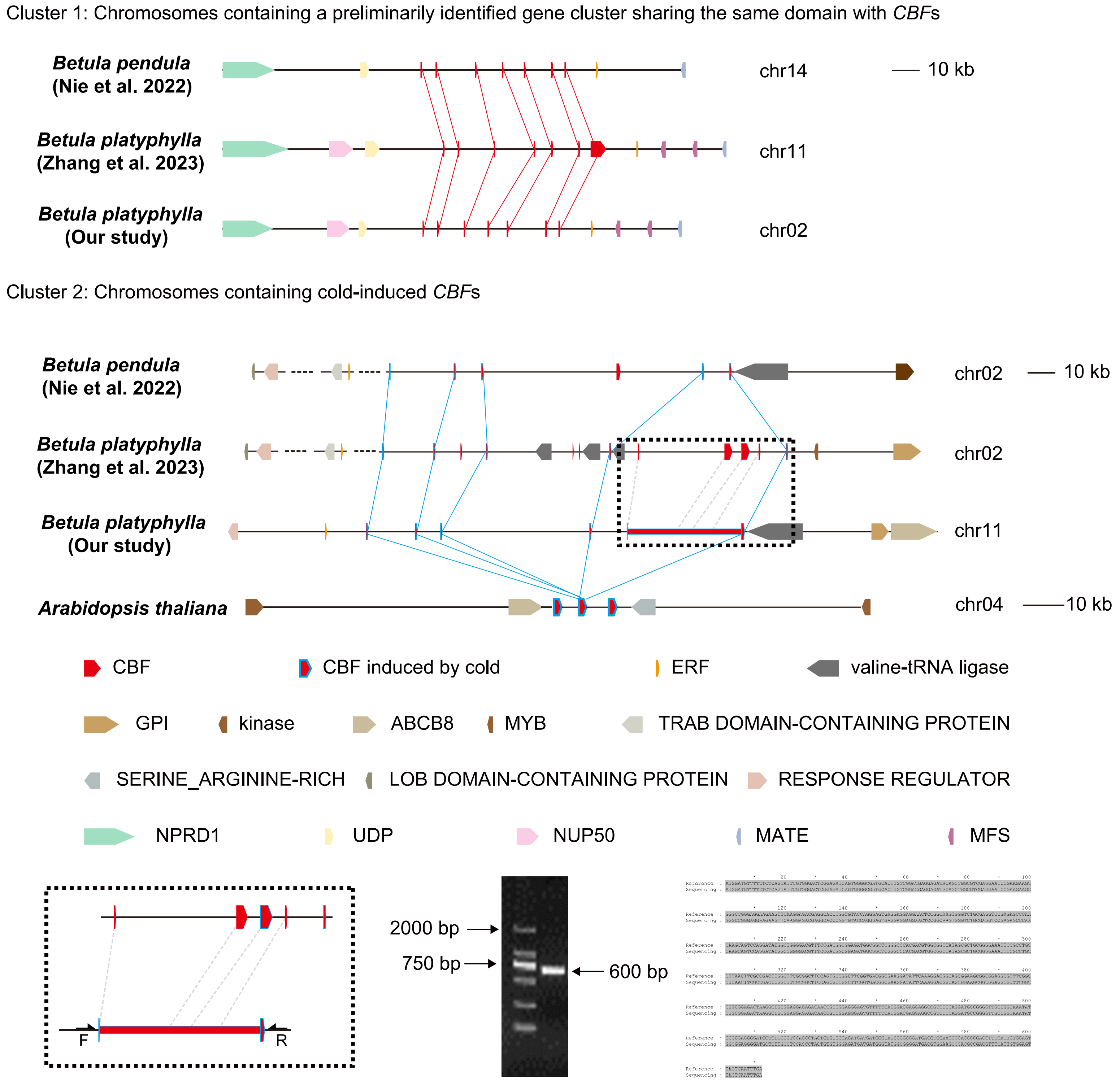

Figure S13 Comparative analysis of *CBF* copy number variation among different birch species.
The upper table summarizes *CBF* gene counts identified in various birch accessions, including total candidate *CBF*s, their chromosomal distribution, and gene IDs of unanchored copies, along with corresponding references. Below, syntenic chromosomal fragments containing putative *CBF*s from three reference genomes are displayed and organized into two clusters. On the chromosomes, red genes represent putative *CBF*s; red genes with blue borders denote cold-responsive *CBF*s supported by experimental evidence from the cited studies; other colors indicate additional annotated genes within these regions. Dashed boxes highlight two genomic regions in Betula platyphylla exhibiting differential cold-responsive CBF compositions. The corresponding primer design sites, PCR validation results, and the corresponding PCR sequencing results are shown in the lower panel.
